# A critical inter‐subunit interaction for the transmission of the allosteric signal in the *Agrobacterium tumefaciens* ADP‐glucose pyrophosphorylase

**DOI:** 10.1002/pro.4747

**Published:** 2023-09-01

**Authors:** Hiral P. Patel, Gabriela Martinez‐Ramirez, Emily Dobrzynski, Alberto A. Iglesias, Dali Liu, Miguel A. Ballicora

**Affiliations:** ^1^ Department of Chemistry and Biochemistry Loyola University Chicago Chicago Illinois USA; ^2^ (UNL‐CONICET), and FBCB Santa Fe Argentina

**Keywords:** ADP‐glucose pyrophosphorylase, allosteric regulation, kinetics, subunit interaction

## Abstract

ADP‐glucose pyrophosphorylase is a key regulatory enzyme involved in starch and glycogen synthesis in plants and bacteria, respectively. It has been hypothesized that inter‐subunit communications are important for the allosteric effect in this enzyme. However, no specific interactions have been identified as part of the regulatory signal. The enzyme from *Agrobacterium tumefaciens* is a homotetramer allosterically regulated by fructose 6‐phosphate and pyruvate. Three pairs of distinct subunit‐subunit interfaces are present. Here we focus on an interface that features two symmetrical interactions between Arg11 and Asp141 from one subunit with residues Asp141 and Arg11 of the neighbor subunit, respectively. Previously, scanning mutagenesis showed that a mutation at the Arg11 position disrupted the activation of the enzyme. Considering the distance of these residues from the allosteric and catalytic sites, we hypothesized that the interaction between Arg11 and Asp141 is critical for allosteric signaling rather than effector binding. To prove our hypothesis, we mutated those two sites (D141A, D141E, D141N, D141R, R11D, and R11K) and performed kinetic and binding analysis. Mutations that altered the charge affected the regulation the most. To prove that the interaction per se (rather than the presence of specific residues) is critical, we partially rescued the R11D protein by introducing a second mutation (R11D/D141R). This could not restore the activator effect on *k*_cat_, but it did rescue the effect on substrate affinity. Our results indicate the critical functional role of Arg11 and Asp141 to relay the allosteric signal in this subunit interface.

## 1 INTRODUCTION

The ADP‐glucose pyrophosphorylase (ADP‐Glc PPase, EC 2.7.7.27) catalyzes a key regulatory step in glycogen biosynthesis in bacteria, and starch biosynthesis in higher plants (Figueroa et al., 2021; Figueroa et al., 2022). The accumulation of glycogen by bacteria may give advantages during starvation periods, providing a stored energy source and carbon surplus (Strange, 1968). ADP‐Glc PPases in Gram‐negative bacteria function as homotetramers (α_4_) of ~50 kDa subunits, whereas in Firmicutes and plants these enzymes are heterotetramers (α_2_δ_2_ or α_2_β_2_, respectively) (Asencion Diez et al., 2013; Ballicora et al., 2003; Cereijo et al., 2016; Cereijo et al., 2018; Figueroa et al., 2022). ADP‐Glc PPase catalyzes the conversion of ATP and glucose 1‐phosphate (Glc1P) to ADP‐glucose (ADP‐Glc) and inorganic pyrophosphate (PPi) in the presence of Mg^2+^. The ADP‐Glc PPase is allosterically regulated by key intermediate metabolites and is involved in carbon assimilation in the host organism (Ballicora et al., 2003; Figueroa et al., 2021; Figueroa et al., 2022). In the case of *Agrobacterium tumefaciens*, ADP‐Glc PPase has been described to be mainly activated by fructose 6‐phosphate (Fru6P) and pyruvate (Pyr) but inhibited by phosphate (Pi) and AMP (Eidels et al., 1970). Both activators can increase the *V*_m_ of the enzyme (“*V*‐type” activation) and the apparent affinity for the substrate ATP (“*K‐*type” activation) (Gomez‐Casati et al., 2001; Segel, 1975; Uttaro et al., 1998).

The similarities between the plant and bacterial enzymes and their evolutionary relationship have helped to understand the structure and function relationship in the whole family. Bacterial enzymes have simpler oligomeric structure and are more convenient in producing recombinant proteins (Ballicora et al., 2003). The plant ADP‐Glc PPase consists of two *small* (S or α) and two *large* (L or β) subunits, where both subunits are needed for a proper physiological function and derive from a common ancestor (Figueroa et al., 2022). Previously, the interaction between subunits has been proposed to be important in the allosteric regulation of the ADP‐Glc PPase. The synergistic interaction between S and L subunits shapes the regulatory properties of the ADP‐Glc PPase in higher plants (Hwang et al., 2005). The areas of the large subunit that participate in tail‐to‐tail and head‐to‐head interactions with the S subunit are important for the allosteric properties of ADP‐Glc PPase from maize (*Zea mays*) endosperm (Georgelis et al., 2009). It was postulated that inter‐subunit interactions might play a role in the allosteric regulation of the potato (*Solanum tuberosum*) tuber enzyme (Kim et al., 2007). There are examples in *Arabidopsis thaliana*, *Ostreococcus tauri*, wheat (*Triticum aestivum*) endosperm, and potato tuber in which the L subunits influence the properties of the S subunit (Ballicora et al., 2005; Crevillen et al., 2003; Ferrero et al., 2018; Figueroa et al., 2018; Kuhn et al., 2009). In some cases, this effect was also observed in hybrids between subunits of different species (Iglesias et al., 2006; Ventriglia et al., 2007). Previous computational studies found that conserved residues in the potato tuber are part of the inter‐subunit interacting regions (Baris et al., 2009; Tuncel et al., 2008).

All the previous literature suggests that specific heteromeric interactions are relevant for regulating ADP‐Glc PPases from photosynthetic eukaryotes. However, we still do not know the mechanism that explains why these interactions are important. In photosynthetic eukaryotes, the more direct description of a specific interaction related to an allosteric effect was found in the potato tuber enzyme. Cleavage of the Cys12 disulfide bond by reduction or removal by mutagenesis yielded a more active enzyme with a higher affinity for the activator 3‐phosphoglycerate (Ballicora et al., 2000), suggesting that the inter‐subunit interaction between S subunits plays a critical role in the allosteric mechanism (Jin et al., 2005). This cleavage of the disulfide bridge is the basis for critical redox regulation observed in several plant tissues (Hendriks et al., 2003; Hou et al., 2019; Tiessen et al., 2002).

In Firmicutes, heterotetramers have different kinetic regulatory properties from the homotetramers (Asencion Diez et al., 2013; Ballicora et al., 2003; Cereijo et al., 2016; Cereijo et al., 2018; Figueroa et al., 2022). This also suggests that the inter‐subunit interactions in those bacteria shape their allosteric properties. However, the information about inter‐subunit interactions is lacking in homotetrameric gram‐negative bacterial enzymes. Pioneer mutagenesis studies combined with recent structural ones hinted that those interactions are important for regulation even in homotetrameric enzymes. Scanning mutagenesis of the *A. tumefaciens* ADP‐Glc PPase has shown that the N‐terminal Arg5 and Arg11 were involved in Pyr activation (Gomez‐Casati et al., 2001). Based on the published structure of the *A. tumefaciens* ADP‐Glc PPase (PDB: 5W6J) Arg11 is located at the interface providing an inter‐subunit interaction with Asp141 (Hill et al., 2019). These two pieces of information indicate that studying the interaction in this interface may provide valuable insights into the allosteric regulation of the enzyme.

Here, we are reporting kinetic properties of different mutants D141A, D141E, D141N, D141R, R11D, R11K and a double mutant R11D/D141R of *A. tumefaciens* ADP‐Glc PPase. This provides relevant information about the contribution of inter‐subunit surface interactions to allosteric regulation. In addition, this research suggests that a better understanding of the subunit interactions can provide insights into designing hybrid ADP‐Glc PPase forms with desired regulatory properties for biotechnological purposes.

## 2 RESULTS

**FIGURE 1:**
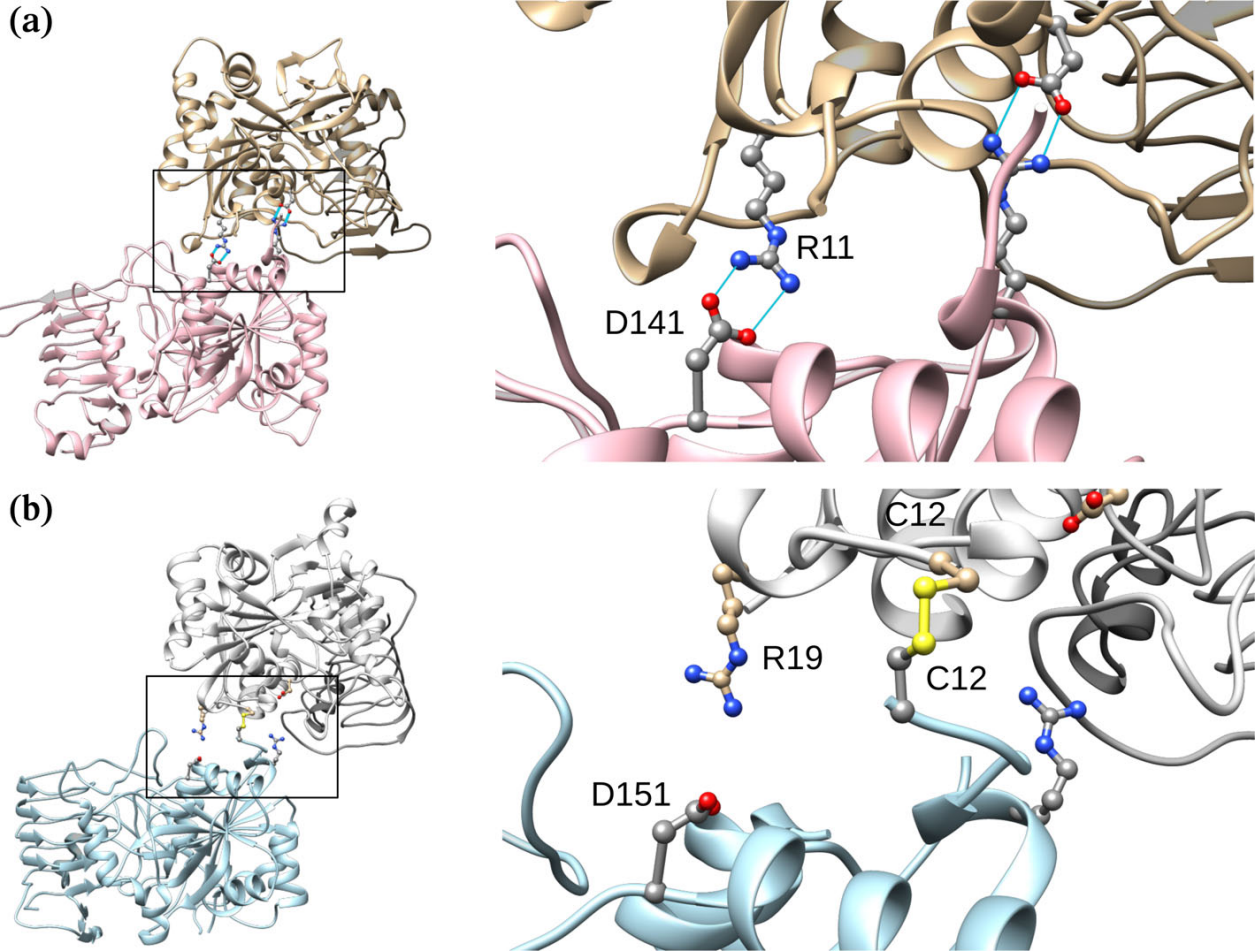
Comparison between the *A. tumefaciens* and potato tuber dimer interface interaction. In panel “a,” the subunit‐subunit interface of the *A. tumefaciens* ADP‐Glc PPase (PDB ID: 5W6J) is compared to the homologous interface of the potato tuber enzyme (PDB ID: 1YP4) in panel “b.” In both cases, the area of the interaction between subunits is zoomed in. In the ADP‐Glc PPase from *A. tumefaciens*, the Arg11 and Asp141 of one subunit make a total of four hydrogen bonds with the Asp141 and Arg11 of the neighboring subunit. In the potato tuber enzyme (inactive form) interface, a disulfide bond between the Cys12 of the two neighboring subunits is shown. The labeled Asp151 and Arg15 in potato tuber structure are the conserved residues homologous to the Asp141 and Arg11 in *A. tumefaciens*, respectively.

According to previous studies, the *A. tumefaciens* ADP‐Glc PPase mutant R11A was desensitized to Pyr activation and was only partially activated by Fru6P (Gomez‐Casati et al., 2001). It was concluded that Arg11 was involved in Pyr activation with the explanation that perhaps it was providing an anionic binding site for the carboxyl group of the ligand (Gomez‐Casati et al., 2001). However, we recently observed in the crystal structure of the *A. tumefaciens* enzyme (PDB: 5W6J) that Arg11, is far from the Pyr binding site, and makes a salt bridge with Asp141 of a neighboring subunit. In the same inter‐subunit interface, Asp141 of the first subunit makes a symmetric interaction with Arg11 of the second subunit. Therefore, in the homo‐tetrameric structure of ADP‐Glc PPase from *A. tumefaciens*, four Arg11 and four Asp141 are making four salt bridges (Figure 1a). Despite these residues not being involved in the binding of the allosteric activators, we hypothesized that not only Arg11, but also Asp141 and the interaction between them are critical for the allosteric effect. We tested this hypothesis with site directed mutagenesis in position 11 and 141 combined with structural, kinetic, and binding analysis.

### 2.1 Gel filtration analysis

**FIGURE 2:**
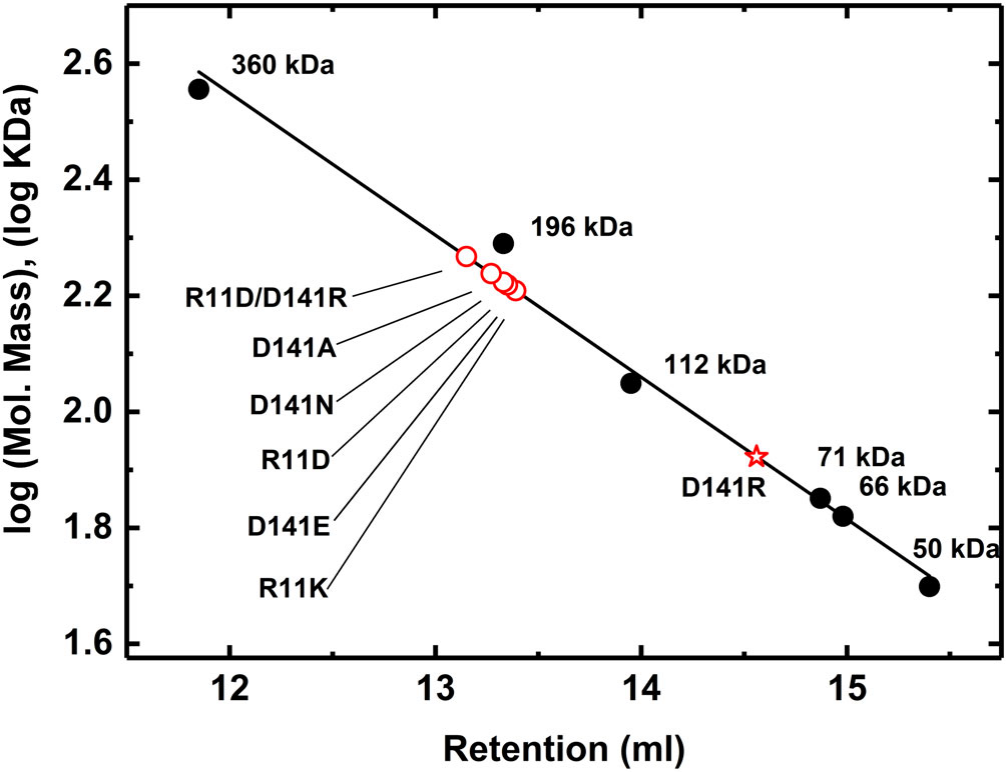
Analytical gel filtration chromatography of ADP‐Glc PPases wild type (WT), and mutants (D141A, D141E, D141N, D141R, R11D, R11K, and R11D/D141R). The gel filtration analysis has been performed using the Superdex™200, 10/300 GL column as indicated in Section 4. The wild‐type ADP‐Glc PPase is a homotetramer, with a molecular weight of ~196 kDa. The markers in solid black circles for this analysis were described in Section 4. The red star represents the D141R enzyme, and the open circles represent R11D, R11K, D141A, D141E, D141N, and R11D/D141R mutant enzymes.

Asp141 and Arg11 are at the interface between two subunits, so we hypothesized that their mutations might disrupt the quaternary structure. For that reason, we analyzed the molecular mass for each of the mutants we produced, which were D141A, D141E, D141N, D141R, R11D, R11K, and double mutant R11D/D141R of *A. tumefaciens* ADP‐Glc PPase. According to an analytical gel filtration study, all the mutant ADP‐Glc PPases were homotetramers like the wild type with the exception of D141R, which appeared to be a homodimer (Figure 2). The retention volume for the wild type was 13.33 mL, whereas for the D141R mutant it was 14.56 mL.

### 2.2 Effect of mutations on the activation by Fru6P and Pyr

**FIGURE 3:**
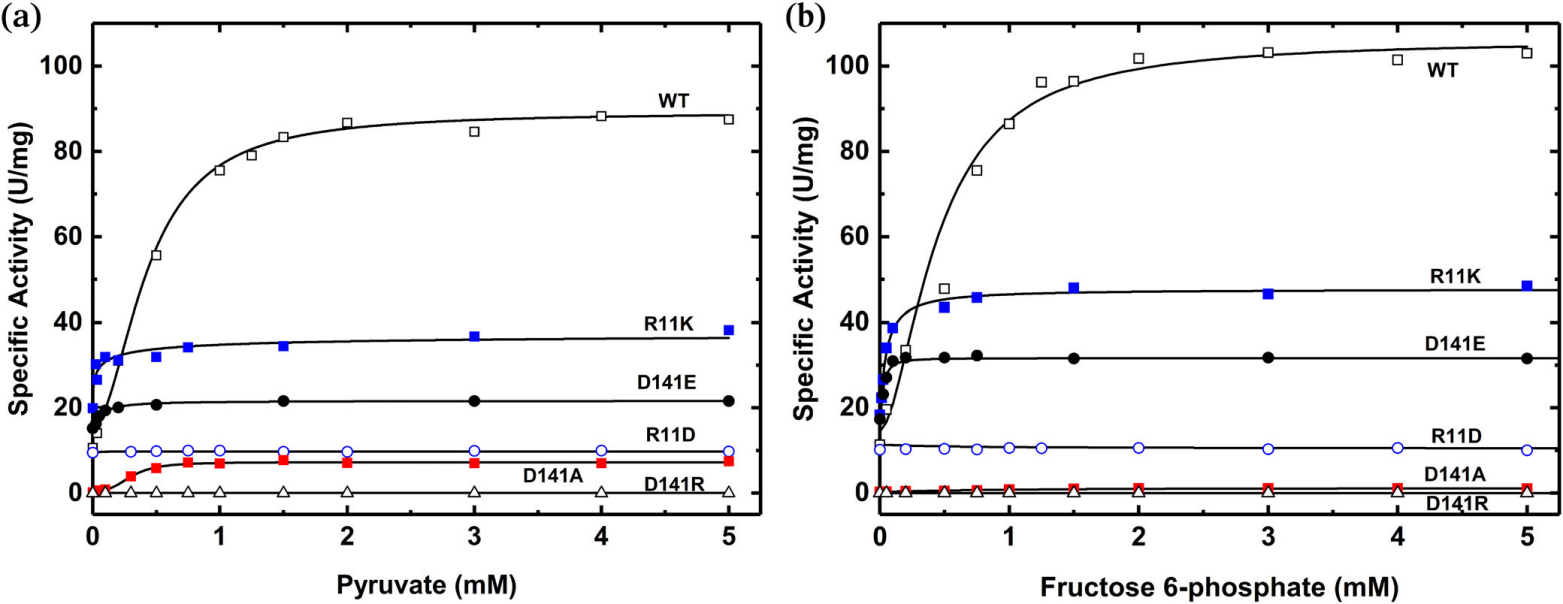
Activator saturation curves of wild type and mutant ADP‐Glc PPase from *A. tumefaciens* at high concentrations of ATP. The effect of Pyr (a) and Fru6P (b) were assayed on the wild type (WT), and D141A, D141E, D141R, R11D, and R11K mutant ADP‐Glc PPases. The assays were performed as described in Section 4 in the presence of 1.5 mM ATP.

To determine the contribution of the inter‐subunit interaction between Arg11 and Asp141 on the enzyme regulation, we first analyzed the activation by Fru6P and Pyr of different mutants at high concentrations of the substrate ATP (1.5 mM). In these conditions, the “*V*‐type” effect of the activator is most dominant since changes in the affinity for ATP will not be emphasized at high concentrations. The altered ADP‐Glc PPases had significant differences when compared to the wild‐type enzyme. Fru6P did not substantially activate the R11D, D141R, and D141N mutants. Mutants R11A and the ones who conserved the charge, D141E, and R11K, were only activated slightly (1.8 to 2.9‐fold) by Fru6P even though they had a higher affinity for the activator (11‐, 6.8‐ and 11.3‐ fold decrease in *A*_0.5_ value). On the other hand, D141A was activated 3.3‐fold by Fru6P but with a reduction in apparent affinity for this activator (2.5‐fold increase in *A*_0.5_ value compared to the wild‐type enzyme;Figure 3, Table S1).

Conservative mutants R11K and D141E were sensitive to activation, whereas R11A, R11D, D141R, and D141N were not (Figure 3, Table S1). R11K and D141E were only slightly activated by Pyr (1.86‐ and 1.47‐fold, respectively) but with an 11‐ and 7.5‐fold decrease in *A*_0.5_ value. On the other hand, D141A was activated 17.8‐fold by Pyr with a similar apparent affinity relative to the wild type. However, the absolute *V*_m_ of D141A was only 7.3 U/mg compared to 87.4 U/mg of the wild‐type enzyme in the presence of Pyr. In fact, the maximum activities (*V*_m_) reached by all the mutants in the presence of saturating concentrations of ATP and activator were significantly lower than the wild type. Only the conservative D141E and R11K reached *V*_max_ of 31.9, and 47.73 U/mg respectively, for Fru6P. Similarly, for Pyr the *V*_m_ reached up to 21.73, and 36.9 U/mg, respectively for D141E, and R11K.

Upon analysis of the ATP apparent affinities of the mutants (see next section below), 1.5 mM of ATP was not high enough for the mutants D141A and D141N to mitigate potential changes in ATP affinity. However, raising the concentration on ATP did not change the overall conclusions. Even at 4 mM ATP, neither Fru6P nor Pyr significantly activated D141N (Figure S1). By raising the concentration of ATP, the mutant D141A had a decrease on the *A*_0.5_ for the Fru6P and Pyr activation (0.84–0.50 mM, and 0.30–0.049 mM respectively). Regardless of the concentration of ATP, the mutations significantly decreased the overall activity of the enzyme and the ability of Fru6P to activate. On the other hand, Pyr could still activate D141A, but not D141N.

### 2.3 Effect of activators (Fru6P, Pyr) on the saturation curves of ATP

To probe whether the synergy between activators and substrates has been altered by side‐chain replacement in sites 11 and 141, we analyzed the effect of the activators Fru6P and Pyr on the kinetic parameters for ATP (Table S2). First, the enzyme assays without activators were analyzed as a baseline condition. In that situation, two mutations that changed the charge of the side chain (D141N and D141R) dramatically decreased *V*_m_ 186‐ and 559‐fold, respectively (Table S2). D141A only decreased *V*_m_ 3.1‐fold and had a 7‐fold higher *S*_0.5_ value for ATP. On the other hand, the conservative mutant D141E behaved like the wild type. The effects of mutations in site 11 were less dramatic than in site 141. Even the radical substitution R11D had a *V*_m_ like the wild type, though with a lower apparent affinity for the ATP (5.3‐fold higher *S*_0.5_ compared to wild type). The R11A mutant was reported with a *V*_m_ 2.7‐fold lower than the wild type and a 2.2‐fold higher *S*_0.5_ value for ATP (Gomez‐Casati et al., 2001). On the other hand, the conservative R11K was slightly more active than the wild type (3.6‐fold higher in *V*_m_) but with lower apparent affinity for ATP. Its *S*_0.5_ value was 4.8‐fold higher than the wild type (Table S2).

The mutations in sites 11 and 141 severely disrupted the effect of Fru6P on the ATP kinetics. The consequence of non‐conservative replacements D141A, D141N, and D141R was that Fru6P lost the ability to regulate *V*_m_ or *S*_0.5_ for the substrate ATP. Fru6P increased the apparent affinity of D141E for ATP like the wild type, but only reached 22% of wild‐type maximum activity. This suggests that charge is critical in site 141. Mutations in site 11 followed a similar pattern. The R11D mutant had the lowest apparent affinity for ATP in the presence of Fru6P. In the R11A enzyme, it was previously described that Fru6P improved the apparent affinity for ATP, but increased *V*_m_ by only ~2‐fold (Gomez‐Casati et al., 2001). The conservative mutation R11K had a similar apparent affinity for ATP but with only 38% of the wild‐type maximum activity. These results show that mutants D141E, R11K, and R11A retained the ability to have a “K‐type activation” by Fru6P, but not D141A, D141N, D141R, and R11D.

The effects of the mutations on the ability of Pyr to regulate ATP kinetics were as severe as the ones analyzed for Fru6P. For instance, D141R and D141N became less sensitive to Pyr altering the ATP affinity. D141A was partially responsive to Pyr, but the *S*_0.5_ for ATP was only lowered 2.3‐fold and *V*_m_ was only increased 2.2‐fold. The mutant D141E was also slightly responsive to Pyr since it decreased the *S*_0.5_ for ATP 2.4‐fold and increased the *V*_m_ 1.7‐fold. The effect of mutations on position 11 depended on the charge introduced. R11D was insensitive to Pyr affecting the ATP affinity. The R11A in the presence of Pyr had only a slightly lower *S*_0.5_ for ATP, compared to the value in the absence of activator (Gomez‐Casati et al., 2001). The conservative mutation R11K allowed Pyr to lower the *S*_0.5_ for ATP 4.4‐fold. These results show that mutants D141E, D141A, R11K, and R11A retained the ability to have a “K‐type activation” by Pyr, but not D141N, D141R, and R11D.

### 2.4 Activation rescue by a secondary mutation (R11D/D141R)

**FIGURE 4:**
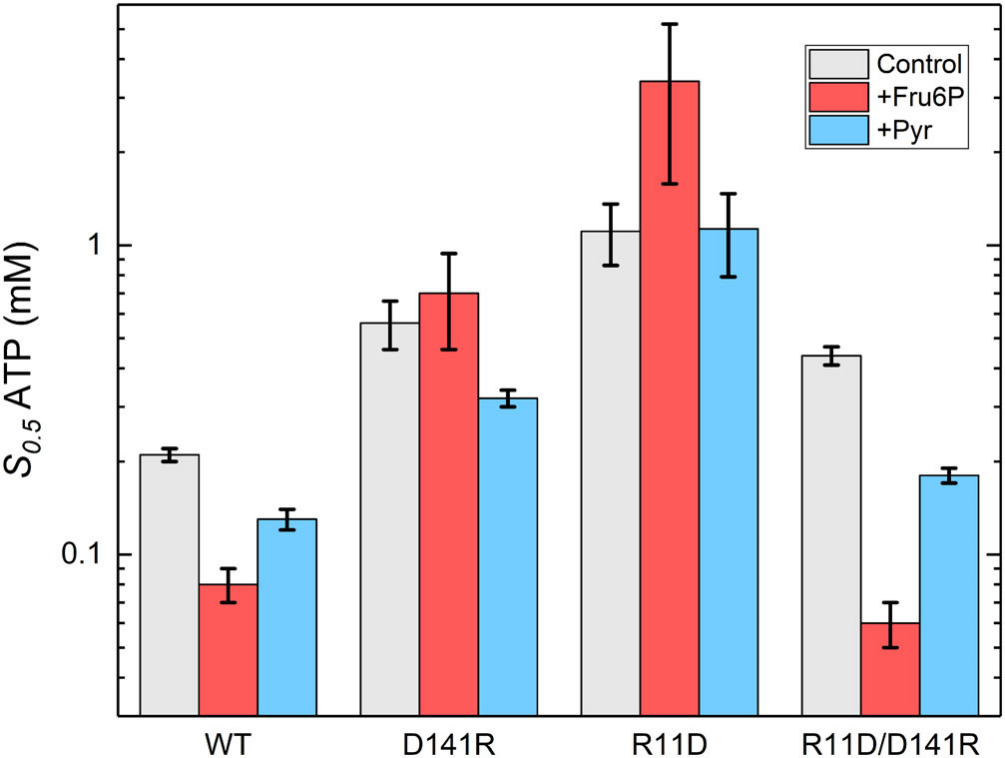
Effect of Fru6P and Pyr on the apparent affinities for ATP in the wild type (WT) and mutant (R11D, D141R, R11D/D141R) *A. tumefaciens* ADP‐Glc PPases. The apparent affinity (*S*_0.5_) for ATP was calculated as described in Section 4. The control is without any activators present, whereas the other +Fru6P, and +Pyr were assayed in the presence of 1.5 mM Fru6P or 1.5 mM Pyr, respectively.

Both single mutations R11D and D141R had drastic effects on activation, whereas the double mutation R11D/D141R rescued the activation by both Fru6P and Pyr. The single mutants R11D and D141R decreased the apparent affinity for the substrate ATP in both the absence and presence of the activators (Fru6P, Pyr; Figure 4). In other words, the activators were unable to decrease the *S*_0.5_ for ATP for those single mutants (Figure 4). Compared to the wild type, the ATP *S*_0.5_ of R11D was 42.4‐ and 8.7‐fold higher in the presence of Fru6P and Pyr, respectively. Similarly, for D141R, the ATP *S*_0.5_ in the presence of Fru6P and Pyr increased up to 8.7‐ and 2.5‐ fold, respectively (Figure 4).

**FIGURE 5:**
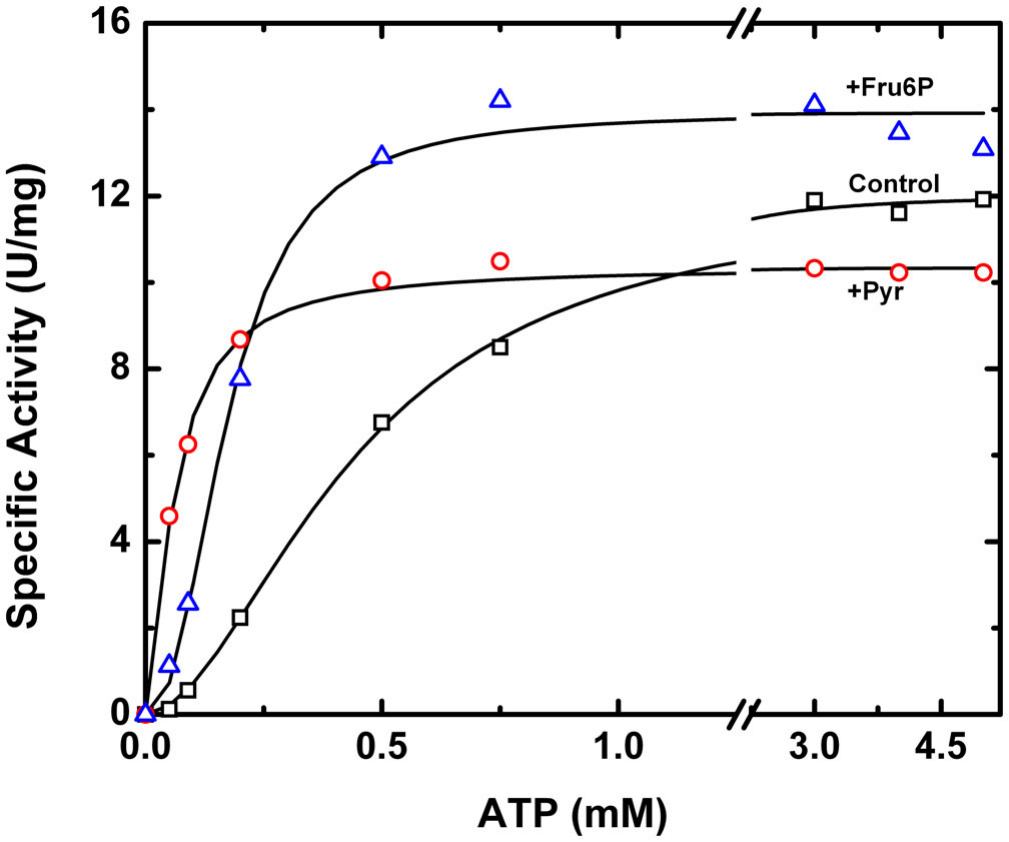
Substrate saturation curve of *A. tumefaciens* ADP‐Glc PPase double mutant R11D/D141R. The ATP saturation curve for the R11D/D141R double mutant ADP‐Glc PPase was conducted in the absence of activator (control), in the presence of 1.5 mM Pyr, and in the presence of 1.5 mM Fru6P. The substrate saturation assays were performed as described in Section 4.

**FIGURE 6:**
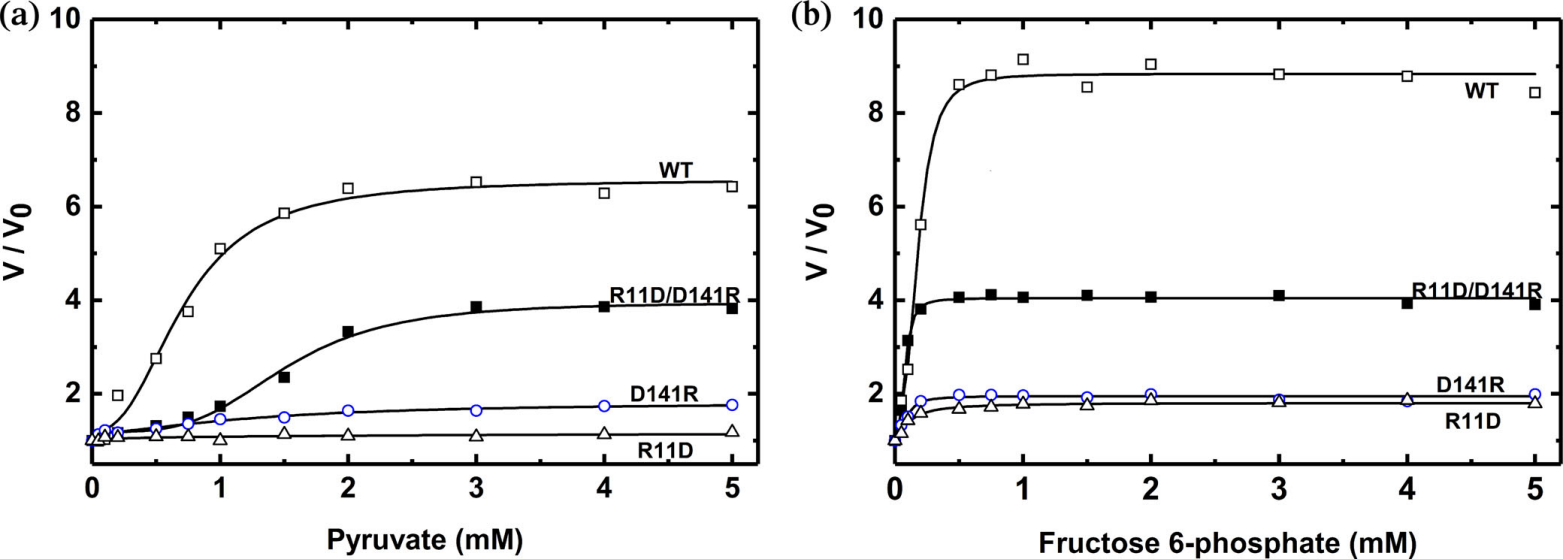
Relative activation curves of wild type and mutant ADP‐Glc PPase from *A. tumefaciens* at sub‐saturating concentrations of ATP. The effects of Pyr (a) and Fru6P (b) were assayed on the wild type (WT), and R11D/D141R, D141R, and R11D mutant ADP‐Glc PPases. The assays were performed as described in Section 4 in the presence of 0.2 mM ATP. *V*_*0*_ is the velocity observed in the absence of activator. In the Pyr saturation curve, *V*_*0*_ for WT, R11D, D141R, R11D/D141R were 8.20, 2.23, 0.006, and 1.97 U/mg respectively. Correspondingly, in the Fru6P saturation curve, *V*_*0*_ for WT, R11D, D141R, R11D/D141R were 8.71, 2.35, 0.007, and 2.17 U/mg respectively.

Without an activator and compared to the wild type, the double mutant R11D/D141R had an identical *V*_m_ (Table S2), and the ATP *S*_0.5_ was only ~2‐fold higher (Figure 4). Meanwhile, in the presence of activators (Fru6P, Pyr), the ATP *S*_0.5_ decreased like the wild type (Table S2, Figure 4, and Figure 5). The ability of D141R to “rescue” the sensitivity of R11D toward activators was evident at low (0.2 mM) sub‐saturating concentrations of substrate (Figure 6). At low ATP substrate concentrations “K‐type” effects are emphasized, alongside “V‐type” effects. In these conditions, the wild‐type enzyme is activated 8.4‐fold by Fru6P (Figure 6). The activities of single mutants R11D and D141R were significantly lower and minimally activated 1.78‐ and 1.98‐fold, respectively. The double mutant recovered a 3.9‐fold activation (Figure 6). A similar “rescue” effect was observed for Pyr, where the wild‐type enzyme, R11D, D141R, and the double mutants were activated 6.4‐, 1.17‐, 1.76‐, and 3.82‐fold, respectively (Figure 6).

### 2.5 Catalytic efficiency

In the absence of activators, the conserved mutations D141E and R11K showed similar catalytic efficiency as wild type (Table 1). In addition, Fru6P increased the catalytic efficiency (*k*_cat_/*S*_0.5_) of these mutants 5.4‐ and 19.9‐fold, respectively. The mutations R11D and D141A decreased 5‐ and 17‐fold the catalytic efficiency in the absence of activators. In addition, these mutations reduced the ability of the activators to regulate the catalytic efficiency (Table 1). Without an activator, the R11A mutant displayed a 6‐fold decrease compared to the wild type, but the activity increased up to 19‐fold in the presence of Fru6P. Conversely, there was little effect by Pyr on the *k*_cat_/*S*_0.5_ of this mutant. On the other hand, the D141N and D141R mutant enzymes lost their sensitivity for activation as well as having a much lower *k*_cat_/*S*_0.5_.

**TABLE 1:** ATP catalytic efficiency of the *A. tumefaciens* ADP‐Glc PPase wild type, and mutants.

Enzyme^a^	Control	+Fru6P^d^	+Pyr^d^
	*k*_cat_ */S*_0.5_ (ATP)^b^ (mM^−1^ s^−1^)	*k*_cat_ */S*_0.5_ (ATP)^b^ (mM^−1^ s^−1^)	*k*_cat_ */S*_0.5_ (ATP)^b^ (mM^−1^ s^−1^)
WT	44.5 ± 1.7	1292 ± 55	549 ± 25
D141E	43.2 ± 2.8	231 ± 11	185.6 ± 7.8
D141A	2.57 ± 0.26	2.27 ± 0.12	10.55 ± 0.38
D141N	0.020 ± 0.001	0.020 ± 0.001	0.020 ± 0.001
D141R	0.040 ± 0.003	0.040 ± 0.001	0.070 ± 0.001
R11K	33.3 ± 1.5	661.3 ± 9.9	141.6 ± 7.7
R11A^c^	7.3 ± 2.4	138 ± 36	14.4 ± 1.8
R11D	9.6 ± 1.2	4.04 ± 0.52	12.4 ± 1.0
R11D/D141R	22.45 ± 0.52	135.1 ± 9.2	64.7 ± 3.3

^a^ Assays were performed as described in substrate saturation as stated under Section 4.

^b^ *k*_cat_/*S*_0.5_ (ATP) was calculated as described in Section 4.

^c^ The results for R11A mutant ADP‐Glc PPase for *A. tumefaciens* were taken from the literature as described in Section 4.

^d^ Concentration of the activator was 1.5 mM.

### 2.6 Binding of activators

**FIGURE 7:**
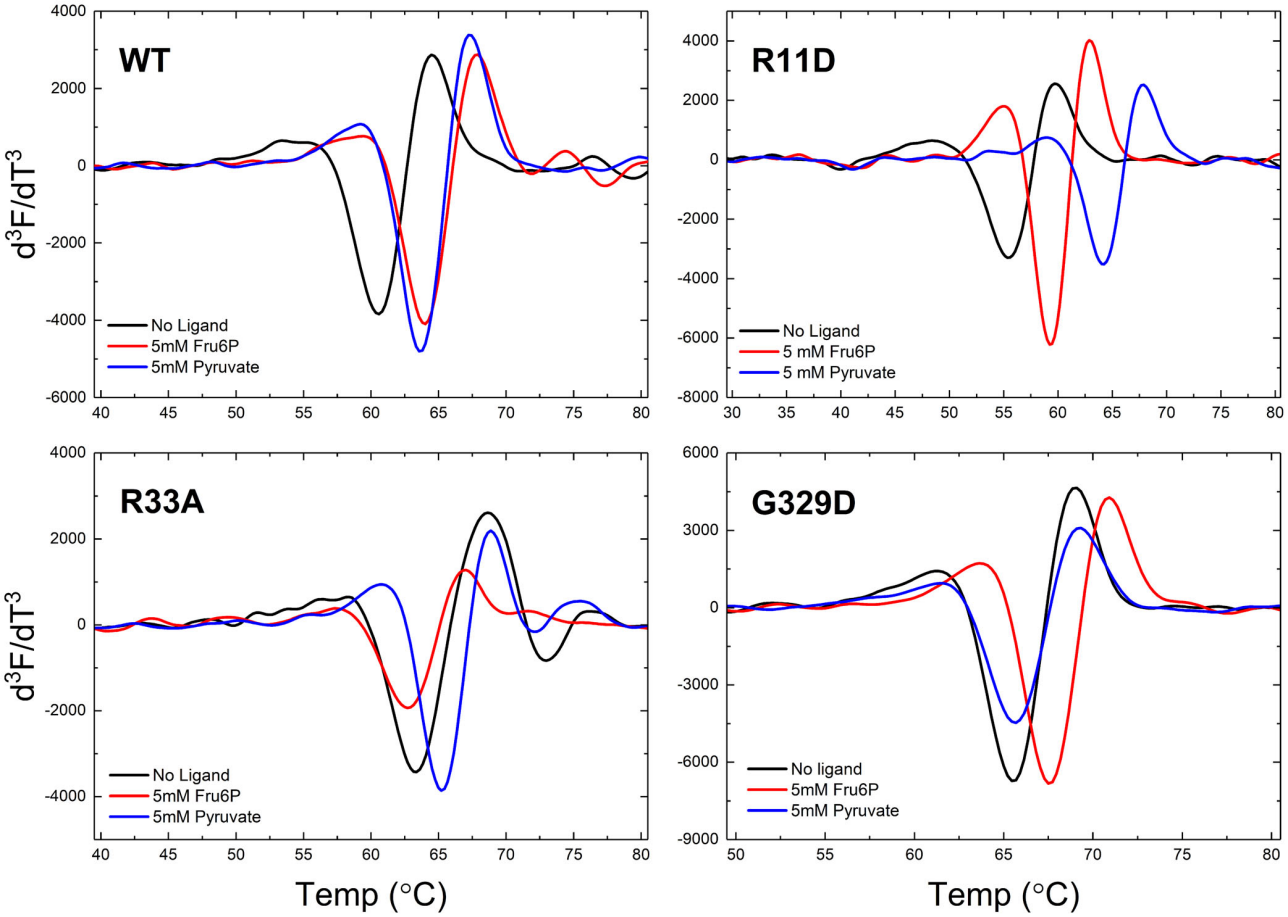
Binding of Fru6P and Pyr. Thermal shifts assays of different enzyme forms in presence of 5 mM Pyr and 5 mM Fru6P were performed as indicated in Section 4. The lowest point of the third derivative of the fluorescence vs Temperature indicates the unfolding or “melting” point (*T*_*m*_) of the protein. For the wild‐type *A. tumefaciens* ADP‐Glc PPase the *T*_*m*_ were 59.8 ± 0.7, 64.2 ± 0.2, and 64.1 ± 0.2 for the control, Fru6P and Pyr respectively. For the R11D mutant the *T*_*m*_ were 55.0 ± 0.1, 58.8 ± 0.1, and 63.5 ± 0.1 for the control, Fru6P and Pyr respectively. For the R33A mutant the *T*_*m*_ were 63.0 ± 0.4, 62.2 ± 0.2, and 65.2 ± 0.1 for the control, Fru6P and Pyr respectively. For the G329D mutant the *T*_*m*_ were 64.8 ± 0.1, 67.5 ± 0.1, and 65.0 ± 0.1 for the control, Fru6P and Pyr respectively.

Mutations of Arg11 and Asp141 dramatically altered the sensitivity to Fru6P and Pyr activation, and R11D was completely insensitive to both. We used thermal shift assays to find whether the effect of the mutation was on the ability of Fru6P/Pyr either to bind or activate the enzyme. Activators of ADP‐Glc PPase bind to the enzyme and stabilize it from thermal denaturation (Bhayani & Ballicora, 2022). Here, we see that Pyr and Fru6P shifted the melting point of the wild‐type *A. tumefaciens* enzyme with a Δ*T*_*m*_ of 4.3 and 4.4°C (Figure 7). As a control, we observed that R33A, which was described to avoid the binding of Fru6P but not Pyr (Gomez‐Casati & Iglesias, 2002) is stabilized by Pyr 2.2°C, but not by Fru6P. On the other hand, G329D, which introduces a carboxylate that blocks the binding of Pyr (Hill et al., 2019), was only stabilized by Fru6P (Figure 7). Both activators stabilized R11D with a Δ*T*_*m*_ of 8.5°C and 3.8°C by the presence of Pyr and Fru6P, respectively (Figure 7). In addition, we measured the dissociation constants (*K*_d_) of Pyr and Fru6P for the wild‐type enzyme and R11D. The *K*_d_ for the wild type was 233 ± 65 μM and 1.51 ± 0.15 mM for Pyr and Fru6P, respectively. The *K*_d_ for R11D was 54 ± 15 μM and 1.82 ± 0.12 mM for Pyr and Fru6P, respectively (Figure S2).

### 2.7 Sequence analysis

According to an alignment of representative ADP‐Glc PPases from diverse species, Asp141 is highly conserved. Out of 57 plant sequences analyzed, 49 sequences have Asp at homologous positions to Asp141 of *A. tumefaciens* ADP‐Glc PPase. The other 8 plant sequences have Asn instead of Asp. Among 134 bacterial sequences, 125 have Asp, 8 have Asn, and only one has Glu. On the other hand, at homologous positions to Arg11, Arg is less conserved compared to Asp at position 141. In plants, 12 sequences have Arg, 22 have Lys, which conserves the positive charge, and 23 have various amino acids. Similarly, in bacteria, 49 sequences have Arg, 32 have Lys, and 53 have other distinct amino acids (Table S3, Figures S2, S3). One of the reasons why an Arg could be less conserved in the homologous position 11 is that other surrogate residues at different positions could replace its role. For instance, in the *Escherichia coli* ADP‐Glc PPase, position 18 (homologous to Arg11 in *A. tumefaciens*) has Leu rather than Arg. However, based on the crystal structure (Cifuente et al., 2016), the interaction of Asp148 (homologous to Asp141 in *A. tumefaciens*) seems to be with an Arg in position 14 rather than 18 (Figure S4). It is very likely that a similar situation happens with other ADP‐Glc PPases from other species. To explore the feasibility of this explanation, we made several homology models of enzymes that have different residues in the homologous position 11 (Figure S8). The model of the enzyme from *Nitrosomonas europaea*, which is only activated by Pyr (Machtey et al., 2012), has a homologous Arg in position 11 interacting with an Asp (Figure S8 panel E). Three other enzymes from *Vulgatibacter incomptus*, *Streptomyces coelicolor*, and *Nitrosomonas* sp. had Arg at different positions from the ones found in *A. tumefaciens* and *E. coli*. In all those models, the Arg is at the proper distance to form a salt‐bridge with an Asp homologous to Asp141 (Figure S8). If a Lys replaces Arg, the same type of interaction could be preserved in *Nostoc sp*. (Figure S8 panel B). Even if that Lys is shifted one position, it is still at a proper distance as we observed in the model of the *Corallococcus llansteffanensis* enzyme (Figure S8 panel C). The fact that a Lys could successfully replace Arg is supported by the mutagenesis data in described in this study (R11K in *A. tumefaciens* had relatively high levels of activity without losing the regulation). All this indicates that there is great flexibility in the N‐terminal region to accommodate a positive charge that will pair with the extremely conserved Asp141 in this enzyme family. For that reason, the conservancy of the interaction is higher than what is predicted by just an inspection of the homologous positions 11 and 141. As it is observed in the phylogenetic analysis (Figure S9), the pairs Arg‐Asp (blue) and Lys‐Asp (light blue) are widespread with some exceptions. As suggested by the models, most of these exceptions could be accounted for by other surrogate and positively charged residues. On the other hand, it is possible that this interaction is absent in some cases. This could be particularly true for subunits in plants in which their role may not be catalytic or binding of activators but to modulate the activity of the other catalytic subunits (Kuhn et al., 2009). However, we found that the enzyme from *Melainabacteria*, which is allosterically activated by sugar phosphates (Glc6P, Fru6P, Man6P; Ferretti et al., 2022), does not have a positively charged residue in the N‐terminal region. Even though this case is in a clear minority, more studies will be needed to find if there is another type of interaction substituting for the one we described in this manuscript.

## 3 DISCUSSION

### 3.1 Structural comparison between plant, and bacterial ADP‐Glc PPase

**FIGURE 8:**
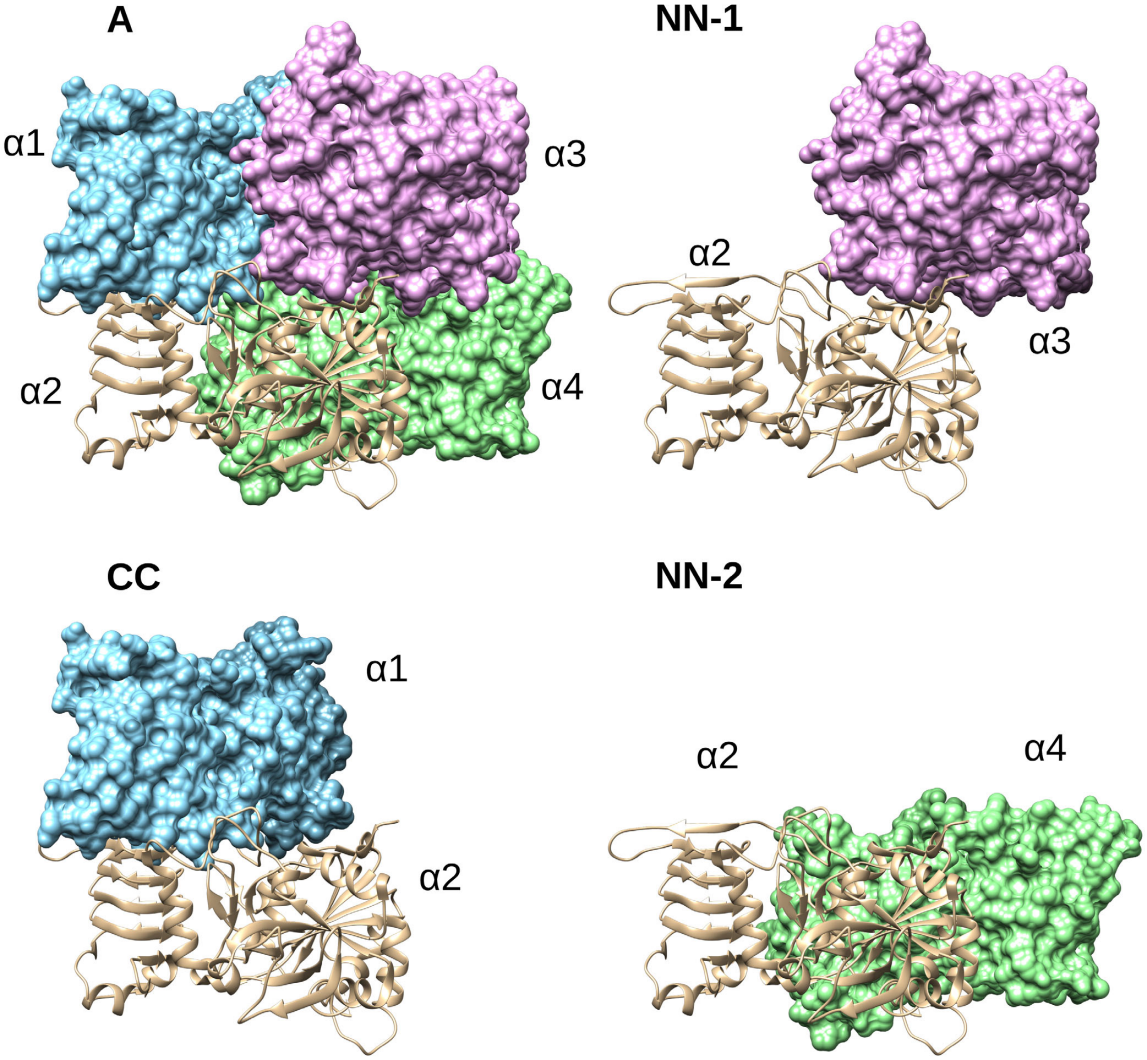
The homotetramer and the dimer interface interaction in ADP‐Glc PPase *A. tumefaciens*. In panel “A,” the homotetrameric *A. tumefaciens* ADP‐Glc PPase (PDB ID: 5W6J) depicts all subunits labeled α1, α2, α3, and α4. In panel “CC,” the C‐terminal to C‐terminal subunit interaction is isolated between subunits α1 and α2. There are two N‐terminal to N‐terminal interfaces, which are depicted in panels labeled “NN‐1” and “NN‐2.”

The homotetrameric bacterial and heterotetrameric plant ADP‐Glc PPases catalyze the same allosterically regulated step in glycogen biosynthesis, and starch biosynthesis, respectively. The similarity allows us to build hypothesis on the plant enzymes based on the knowledge gathered with simpler oligomeric bacterial forms (Ballicora et al., 2003; Figueroa et al., 2022). The study of the specific amino acids involved in inter‐subunit interactions provides important insights into the structural stability and regulation of the enzyme family (Georgelis et al., 2009). The ADP‐Glc PPase from *A. tumefaciens* functions as a tetramer, which can be described as a dimer of dimers. For convenience, we divided the dimer subunit interactions into three groups: NN‐1 (with interactions between both N‐termini from subunits α2‐α3 or α1‐α4), NN‐2 (N‐termini interactions from α2‐α4 or α1‐α3 subunits), and CC (C termini from α2‐α1 or α3‐α4) (Figure 8; Hill et al., 2019). The N‐terminal‐to‐N‐terminal interaction has two possible contacts (NN‐1 and NN‐2), whereas the C‐terminal has only one. In the past, studies of different residues at interfaces CC and NN‐2 highlighted the importance of subunit interaction in regulation (Georgelis et al., 2009; Greene & Hannah, 1998; Hwang et al., 2005; Salamone et al., 2002).

The dimer interactions NN‐2, and CC from *A. tumefaciens* are identical to the described “head‐to‐head” (NN‐2 in our nomenclature) and the “tail‐to‐tail” interaction (CC), respectively, between the large subunit (SH2) and the small subunit (BT2) from maize endosperm (Georgelis et al., 2009). Overall, these interactions of maize endosperm were proposed to be significant for allosteric properties, and specifically for the affinity for allosteric effectors (Georgelis et al., 2009). It has been observed that changes in the CC interface could lead to altered allosteric properties in the potato tuber enzyme. For instance, random mutants S_devo330_ (Y315C, L378S) and S_devo355_ (Y315C, K295E) enhanced affinity for the activator 3‐phosphoglycerate and increased resistance to Pi inhibitor (Salamone et al., 2002). Here, analysis of the crystal structure of the potato tuber small subunit (Jin et al., 2005), leads to the conclusion that the common residue in the mutants, Y315, (residue Y317 in the crystal structure nomenclature) makes an “edge to face” π–π interaction (Anjana et al., 2012). This interaction links two different subunits in the CC dimer interface (Figure S10). According to mutagenesis studies, it was proposed that the synergy between large and small subunits is important for the regulation of the enzyme (Hwang et al., 2005; Salamone et al., 2002).

Our studies here are mainly about the NN‐1 dimer interface of *A. tumefaciens* ADP‐Glc PPase. As a reference, in the NN‐1 dimer interface in the potato tuber enzyme, there is a disulfide bond between Cys12 of subunits A, and A' (α subunits only because the β subunits with no conserved Cys12 residue do not share a disulfide bridge; Jin et al., 2005). Interestingly, the NN‐1 interface of the *A. tumefaciens* enzyme does not have a disulfide bond, but instead has two salt bridges. This difference hinted toward the importance of the salt bridges based on the previous knowledge collected on the potato tuber enzyme. Cleavage of the disulfide bond between Cys12 residues by reduction or removal by mutagenesis activates the potato tuber enzyme (Ballicora et al., 2000). Overall, the disulfide bond between α subunits of potato tuber keeps the two catalytic dimers in their inactive form (Ballicora et al., 2000). For that reason, the crystal structure study of the potato tuber enzyme supports the hypothesis that the inter‐subunit interaction NN‐1 between the α‐subunits is an important part of the allosteric mechanism (Jin et al., 2005).

According to our analysis, in the crystal structure of the *A. tumefaciens* ADP‐Glc PPase (Hill et al., 2019), at the interface NN‐1 Arg11 of α2 makes a salt bridge with Asp141 of the neighboring subunit α3. At the same interface, Asp141 of the α2 subunit makes another salt bridge with Arg11 of the α3 subunit (Figure 1a). Similarly, the other dimer α1‐α4 has another two salt bridges between Arg11 and Asp141 at a similar NN‐1 type interface. Therefore, in the homotetramer structure of the ADP‐Glc PPase of *A. tumefaciens*, four Arg11 and four Asp141 make four salt bridges, in total. The conserved Asp151 and Arg19 of the potato tuber small subunit (Jin et al., 2005) are in the same sequence position and homologous to the Asp141 and Arg11 of *A. tumefaciens* ADP‐Glc PPase (Figure 1b). This analysis leads us to the hypothesis that putative salt bridge interactions at the interface at the potato tuber ADP‐Glc PPase enzyme may have a related role. Regarding the conformational changes occurring upon reduction of the potato tuber ADP‐Glc PPase, it is important to note that we currently lack direct structural evidence. The only available crystal structure is in the oxidized form of the homotetramer (α_4_), likely because the reduced form has been shown to be less stable (Ballicora et al., 1999). Furthermore, it is important to note that the major native form of the enzyme is α_2_β_2_, consisting of two α (“small”) subunits and two β (“large”) subunits in the quaternary structure. While the NN‐1 interface is expected to be between the α subunits due to the presence of the disulfide bridge (Jin et al., 2005) and energy calculations (Tuncel et al., 2008), the presence of the β (large) subunit might slightly influence the conformation of the α subunits and their relative orientations. To gain a deeper understanding of the nature of the residue pairs interacting in the reduced and oxidized forms, further investigations, including biophysical and mutagenesis studies, should be conducted at the NN‐1 interface in plant ADP‐Glc PPases.

### 3.2 The effect of mutations at the interface NN‐1

Mutations of the Arg11 and Asp141 residues affect the allosteric regulation as well as the structural stability of the ADP‐Glc PPase. According to our quaternary structure analysis, the D141R mutant was a homodimer, in contrast with other homotetramer mutants (Figure 2). In the mutant D141R, we can say that the two positive charges facing each other (Arg11 and Arg141) destroyed the interface interaction and resulted in a homodimer. The residues Arg11 and Asp141 interact in the inter‐subunit surface holding the subunits in a proper position to become activated. We can hypothesize that after cleaving the disulfide bond between Cys12 in the potato tuber ADP‐Glc PPase, the conserved residues Arg19 and Asp151 come together to stabilize the structure (Figure 1b). The conserved mutations R11K, and D141E maintained the enzyme sensitive to the activation by Fru6P and Pyr. This indicates that the charges in these residues are critical.

At a sub‐saturating concentration of ATP (0.2 mM), neither of the activators had significant effects on mutants R11D and D141R. Pyr only slightly increased D141R activity (1.76‐fold), which was similar to how Fru6P activated R11D and D141R (1.78‐ and 1.98‐fold; Figure 6). The activation by Pyr and Fru6P are much lower than the wild‐type enzyme, which were 6.4‐fold and 8.4‐fold, respectively (Figure 6). Even at higher concentrations of ATP (1.5 mM), still Fru6P and Pyr do not seem to activate the mutant enzymes R11D and D141R significantly (Figure 3). In contrast to the single mutants, at sub‐saturating concentrations of ATP, the double mutant R11D/D141R partially restores the activation by Pyr and Fru6P (3.8‐ and 3.9‐fold), respectively (Figure 6). These results indicate that single mutations R11D and D141R dramatically lowered the effect of the activators in the enzyme. The mutant R11D is particularly interesting because it was completely insensitive to Fru6P and Pyr activators. However, it retained the ability to bind both (Figure 7). The dissociation constants for Pyr and Fru6P were 54 ± 15 μM and 1.82 ± 0.12 mM, respectively (Figure S2). At those concentrations, no activation was detected for R11D (Figure 3). That means that even when the mutant has the activator bound, no activation is triggered, indicating that it lost the ability to communicate the activation signal from the regulatory to the active site. A similar scenario was previously found in two different loops of the *A. tumefaciens* enzyme (near Gln67 and Trp106, respectively), which indicated that those were responsible for triggering the allosteric signal for the sugar phosphate activator Fru6P (Asencion Diez et al., 2020). The same role for those loops was also assigned in the *E. coli* and the potato tuber enzyme (Figueroa et al., 2013) for the activation by FBP and 3‐phosphoglycerate, respectively. However, those homologous loops do not seem to be the main ones responsible for triggering the activation by Pyr in *E. coli* and *A. tumefaciens*. That is the basis for proposing that the two activators (Pyr and the sugar phosphates) do not trigger the same allosteric signal. Here, mutations at the position Arg11 and Asp141 in *A. tumefaciens* diminished or blocked the allosteric signal for both activators and their apparent affinity. In contrast, the double mutant enzyme R11D/D141R restores the analogous effects of activators compared to the wild type. Overall, we can say that it is not necessary to have an Arg at position 11 or Asp at position 141 to relay the allosteric signal, but the charge of these residues is critical.

The allosteric control of ADP‐Glc PPases is critical to manipulate the physiology of glycogen production in bacteria and starch in plants. For that reason, to rationally engineer this enzyme, it is essential to understand the allosteric mechanism and how the signal is transmitted. Here, we found critical inter‐subunit interactions in the *A. tumefaciens* ADP‐Glc PPase that allow the activators to trigger the activation after binding to the enzyme. The role of this inter‐subunit interaction in enzymes from other species that are activated by different metabolites could contribute to understanding the evolution of this functional feature. This is particularly important in certain plant enzymes where reduction of disulfide bonds in this area play a critical regulatory role. This will provide insights into the manipulation of the enzyme for increased starch production.

## 4 MATERIALS AND METHODS

### 4.1 Materials

Biochemicals used for enzyme assays, yeast pyrophosphatase, and bovine serum albumin were from Sigma‐Aldrich (St. Louis, MO). *Escherichia coli* BL21 (DE3) cells were purchased from New England BioLabs (Ipswich, MA). Bacterial growth media and antibiotics were provided by IBI Scientific (Pittsburgh, PA). As gel filtration markers, *Nitrosomonas europaea* sucrose synthase (Wu et al., 2015), *E. coli* glycogen synthase (Yep et al., 2004), and *Bacillus subtilis* Gab‐R (Edayathumangalam et al., 2013) were purified and obtained as indicated previously. All the other chemicals were of the highest quality available.

### 4.2 Site‐directed mutagenesis

The PCR for site‐directed mutagenesis was performed using a Q5 Site‐Directed Mutagenesis Kit (New England Biolab). The pBLH1 vector (a pET28c derivative) containing the *A. tumefaciens* ADP‐Glc PPase was used as a template (Hill et al., 2019). The oligonucleotides for mutations were designed using BioEdit software and synthesized by Integrated DNA Technologies (IDT) (Hall, 1999). The pairs of primers that were used for generating the mutations D141A, D141E, D141N, D141R, R11D, and R11K in *A. tumefaciens* ADP‐Glc PPase were as follows. For D141A, forward, 5'‐CAT ATT TAC AAA ATG GCC TAC GAA TAC‐3′ and reverse 5′‐GTC GCC GGC CAG AAT GAC CAT‐3′; For D141E, forward, 5′‐ CAT ATT TAC AAA ATG GAG TAC GAA TAC ATG CTG C‐3′ and reverse 5′‐GTC GCC GGC CAG AAT GAC CAT‐3′; For D141N, forward, 5′‐ CAT ATT TAC AAA ATG AAC TAC GAA TAC ATG CTG C‐3′ and reverse 5′‐GTC GCC GGC CAG AAT GAC CAT‐3′; For D141R, forward, 5′‐ CAT ATT TAC AAA ATG CGC TAC GAA TAC ATG CTG −3′ and reverse 5′‐GTC GCC GGC CAG AAT GAC CAT‐3′; For R11D, forward, 5′‐GCG GAT GAT GCA ATG GCC TAT GTC CTC‐3′ and reverse 5′‐CAA AGG CTG AAC TCT TTT TTC CGA CAT‐3′; For R11K, forward, 5′‐GCG AAG GAT GCA ATG GCC TAT GTC CTC G‐3′ and reverse 5′‐CAA AGG CTG AAC TCT TTT TTC CGA CAT‐3′. The mutations were verified by genetic sequencing performed by the University of Chicago Comprehensive Cancer Center DNA Sequencing and Genotyping Facility in Chicago, Illinois.

### 4.3 Expression and purification

The wild type and the mutants of the *A. tumefaciens* ADP‐Glc PPase were expressed in transformed *E. coli* BL21 DE3 cells. Cells were transformed with plasmid pBHL1 encoding for the wild‐type enzyme with a His‐Tag (Hill et al., 2019). Mutant enzymes were encoded by derivatives of pBHL1 obtained as described above. Transformed cells were grown to an OD_600_ between 1.1–1.3 at 37°C and cooled on ice. The culture was induced with 0.4 mM isopropyl β‐D‐1‐thiogalactopyranoside (IPTG) at 25°C for 16 h with shaking at 250 rpm. The cells were harvested by centrifugation and sonicated in buffer C (50 mM HEPES pH 7.5, 10% glycerol, 200 mM NaCl, and 10 mM imidazole). Crude extracts were loaded onto a pre‐equilibrated 5 mL His‐Trap FF column (Ni^2+^ Sepharose column) and eluted with a linear gradient 0–50% of buffer E (50 mM HEPES pH 7.5, 10% glycerol, 200 mM NaCl, and 750 mM imidazole). Fractions were analyzed by SDS‐PAGE and activity assays. Active ones were pooled, concentrated in buffer E, and stored at −80°C in aliquots. The concentrated proteins were further purified using an equilibrated gel filtration column (Superdex™200, 10/300 GL) with buffer X (50 mM HEPES pH 7.5, 5% glycerol, and 200 mM NaCl). The list of the markers that were used is as follows; *N. europaea* sucrose synthase (360 kDa); *A. tumefaciens* ADP‐Glc PPase (196 kDa); *B. subtilis* Gab‐R (112 kDa); yeast pyrophosphatase (71 kDa); bovine serum albumin (66 kDa); and *E. coli* glycogen synthase (50 kDa).

### 4.4 Enzyme assay

ADP‐Glc PPase activity was measured by the production of pyrophosphate (PPi) as previously described (Figueroa et al., 2011). After hydrolysis of PPi with pyrophosphatase, the phosphate generated is assayed as indicated (Fusari et al., 2006). The Malachite Green‐Ammonium Molybdate‐Tween 20 (MG‐AM‐T20) solution was used in the enzyme assay to stop the reaction and detect phosphate (Fusari et al., 2006). The total reaction volume was 50 μL and was performed in polystyrene flat‐bottom microplates for 10 min until stopped with MG‐AM‐T20. The absorbance was measured at 620 nm in a Multiskan Ascent reader. The unit of enzyme activity (U) is defined as 1.0 μmol of PPi formed per minute.

*Substrate saturation assay*: The reaction mixture contained 50 mM HEPES (pH 7.5), 14 mM MgCl_2_, 1.5 mM Glc1P, 5 U/mL inorganic pyrophosphatase, and 0.2 mg/mL bovine serum albumin (BSA) in presence of varying concentrations of substrate ATP. Similar reactions were performed in the presence of 1.5 mM of either Fru6P or Pyr (activators).

*Activator saturation assay*: The reaction mixture for this reaction contained 50 mM HEPES (pH 7.5), 7 mM MgCl_2_, 1.5 mM Glc1P, 1.5 mM ATP, 5 U/mL inorganic pyrophosphatase, and 0.2 mg/mL BSA, in presence of varying concentrations of either Fru6P or Pyr. This condition was considered “saturating concentrations” of substrate. To analyze the effect of the activators in “sub‐saturating concentrations” of substrate, the concentration of ATP was 0.2 mM.

### 4.5 Kinetics

Modified Hill equations, $v=V_{m}\;S^{n_{H}}/\left(S_{0.5}^{n_{H}}+S^{n_{H}}\right)$ or $v=V_{0}+\left(V_{m}-V_{0}\right)\;A^{n_{H}}/\left(A_{0.5}^{n_{H}}+A^{n_{H}}\right)$ were used to fit the kinetic data as indicated previously (Bhayani et al., 2019). Velocity is indicated by v, S is the concentration of substrate, A is the concentration of the activator, and *n*_H_ is the Hill coefficient. The parameter *S*_0.5_ indicates the concentration of substrate needed to reach 50% of the maximal velocity (*V*_m_). In activation curves, where the basal activity in absence of activator (*V*_0_) is not zero, *A*_0.5_ values indicate the concentration of activator needed to give 50% of the maximal activation response (*V*_m_−*V*_0_). The rate constant *k*_cat_ was calculated per each monomer from *V*_m_ assuming a molecular mass of 50 kDa. All kinetic assays were performed at least in duplicate with reproducibility of parameters within ±10%. Kinetic parameters of the *A. tumefaciens* ADP‐Glc PPase mutant R11A were obtained from literature.

### 4.6 Thermal shifts assays

Thermal shift assays were performed as described (Hill et al., 2019) using the QuantStudio™ 3 Real‐Time PCR System (Thermo Fisher Scientific) and QuantStudio™ Design and Analysis Software v1.5.1. The final volume for the assay was 20 μL and contained 50 mM HEPES (pH 8.0), SYPRO Orange Dye (Sigma‐Aldrich) (4×) and 0.02 mg/mL purified protein, in the absence or presence of 5 mM Fru6P, and 5 mM Pyr. A control with no protein was also performed for all the samples. A continuous temperature increases from 25.0 to 95.0°C was scanned at a ramp increment of 0.1°C per second. Scans were run in triplicates and averaged. The unfolding temperatures (*T*_*m*_) of the proteins were measured using the minimum of the third derivative of the scanned fluorescence vs. temperature (d^3^F/dT^3^) (Bhayani & Ballicora, 2022).

### 4.7 Data processing of thermograms

Thermal Shift assays were used to study the relationship between the melting temperature ($T_{m}$) of a protein and the logarithm of the ligand concentration using the following equation (Bhayani & Ballicora, 2022).

$$y=T_{m_{0}}+A\;\log_{10}\left(1+10^{x}/K_{d}\right).$$

In this equation, y is $\;T_{m}$, and x is the logarithm of the ligand concentration. The parameter $T_{m_{0}}$ is the melting point of the protein in absence of ligand, $K_{d}$ is the dissociation constant of the ligand, and A is a parameter grouping other thermodynamic parameters and constants that do not change significantly in the range of temperatures analyzed (Bhayani & Ballicora, 2022). The data was fitted to an equation using Origin™ 2017 and the Levenberg–Marquardt algorithm, to find the optimum parameters $T_{m_{0}}$, A, and $K_{d}\;$ (Bhayani & Ballicora, 2022). To ensure robustness and reliability, all thermal shift assays were conducted in triplicate. The parameters obtained from these replicates exhibited reproducibility within ±10%.

### 4.8 Sequence alignment and phylogenetic analysis

Sequences coding for bacterial ADP‐Glc PPases were downloaded from the NCBI database (http://www.ncbi.nlm.nih.gov/). They were filtered to remove duplicates and near duplicates (i.e., mutants and strains from same species). Sequences were chosen to represent major taxonomic groups of bacteria and plants. Sequences were classified into different groups using taxonomic data provided by the NCBI as depicted in Figure S5. The ClustalW multiple sequence alignment process (Thompson et al., 1994) was performed then manually refined with the BioEdit 7.0 program (Hall, 1999). A manual inspection was performed to guarantee that all known conserved regions (catalytic residues etc.) were properly aligned. Phylogenetic and construction of tree were conducted using MEGA version 11 (Tamura et al., 2021). The FigTree 1.4.4 program (https://github.com/rambaut/figtree/) was used to arrange the complete phylogenetic tree.

### 4.9 Homology modeling and structural analysis

Modeling of the ADP‐Glc PPase homotetrameric structure for the *V. incomptus*, *C. llansteffanensis*, *Nostoc* sp., *S. coelicolor*, *Nitrosomonas* sp., and *N. europaea* ADP‐Glc PPase was performed using the program Modeller 9.11 (Sali & Blundell, 1993) as described before with minor modifications (Cereijo et al., 2018). The structure was modeled using the structure of the homologous enzymes from *A. tumefaciens* (PDB code 5W5R) and the small subunit from *S. tuberosum* (1YP3). To check the feasibility of the interaction between Asp141 or their homologous residues, and the putative positively charged counterpart, Modeller was used to force the distance between them to 2.8 Å with an error of 0.03 Å. The reliability of the models were evaluated using the program ProSA‐web (Wiederstein & Sippl, 2007). The structure analysis and protein visualization were performed using UCSF Chimera (Pettersen et al., 2004).

## CONFLICT OF INTEREST STATEMENT

The authors declare no conflicts of interest.

## Supporting information


**Figure S1.** Activator saturation curves of *A. tumefaciens* ADP‐Glc PPase mutants D141A and D141N at 4 mM of ATP. The effect of Pyr and Fru6P were assayed on the D141A and D141N mutant ADP‐Glc PPases. The assays were performed as described in Materials and Methods in the presence of 4 mM ATP.
**Figure S2.** Binding analysis for the *A. tumefaciens* ADP‐Glc PPase WT and R11D. Panel A and B are for the wild‐type enzyme, and panel C and D for the mutant R11D. Thermals shifts of the melting points (*T*_*m*_) at different concentration of activators Fru6P and Pyr have been performed as indicated in Material and Methods. Fitting of the data to obtain dissociation constants (*K*_d_) has also been described in Material and Methods.
**Figure S3.** Sequence alignment for several species of plants and bacteria used for phylogenetic analysis. Highlighted are the homologous positions to Arg11 (region 1) and Asp141 (region 2) in Agrobacterium tumefaciens. Le column represents the code used in the phylogenetic tree (Figure S9).
**Figure S4.** Sequence alignment for several species of photosynthetic eukaryotic ADP‐glucose pyrophosphorylase subunits. Highlighted are the homologous positions to Arg11 and Asp141 in *Agrobacterium tumefaciens*.
**Figure S5.** Sequences of ADP‐glucose pyrophosphorylases used for phylogenetic analysis. Sequences, GI numbers, accession codes, and taxonomy were obtained as indicated in Materials and Methods.
**Figure S6.** Sequences of ADP‐glucose pyrophosphorylases from plants used to analyze the conservancy of residues. Sequences, GI numbers, and accession codes were obtained as indicated in Materials and Methods.
**Figure S7.** Dimer interaction between D148 and R14 in the *E. coli* ADP‐Glc PPase. Residues D148 and L18 are in homologous position to D141 and R11 in *A. tumefaciens* ADP‐Glc PPase, respectively. Figure has been constructed using the coordinates from the *E. coli* ADP‐Glc PPase structure (PDB: 5L6S) as indicated in Materials and Methods.
**Figure S8.** Modeling of the N‐terminal domain interaction in representative ADP‐glucose pyrophosphorylases Enzymes in panel A to F were selected to represent different type of possible interactions in different species. These interactions are with the Asp homologous to Asp141 in *A. tumefaciens* Enzyme. The sequences are also aligned to the structures from *A. tumefaciens*, *E. coli*, and *S. tuberosum* of which crystal structures are displayed in Figure 1 and Figure S7. Arrows in the bottom table represent the homologous positions to the Arg11 and Asp141 in *A. tumefaciens*.
**Figure S9.** Phylogenetic tree of ADP‐glucose pyrophosphorylases from bacteria and photosynthetic eukaryotes. Sequences of the glgC gene products from bacteria and homologous from photosynthetic eukaryotes were collected and the tree was built as described in Materials and Methods. In the tree, it is the taxonomy information matching colors with the id codes for each individual gene. Blue circles indicate enzymes with Arg and Asp in homologous positions 11 and 141 respectively (*A. tumefaciens* nomenclature). Light blue circles have Lys and Asp, Green does not have an Arg or Lys in position 11, but has Asp in position 141. Purple has an Arg in position 11, but no Asp in 141. Red has neither of those residues conserved. White stars depict variants with crystal structures analyzed in this work. Black stars are forms that were modeled in Figure S8.
**Figure S10.** Interaction at the CC interface of the homotetrameric potato tuber small subunit ADP‐Glc PPase. Depicted residues Tyr317 from the light blue subunit interact with the Tyr312 subunit in pink. The interaction is “edge to face” as described in Discussion.

**Table S1.** Kinetic parameters for the activators of the *A. tumefaciens* ADP‐Glc PPase wild type, and mutants.
**Table S2.** Kinetic parameters for the substrate (ATP) of ADP‐Glc PPase wild type, and mutants.
**Table S3.** Conservancy of residues Arg11 and Asp141 in various plant and bacterial ADP‐glucose pyrophosphorylases.
