# Supplementary material for: A critical inter‐subunit interaction for the transmission of the allosteric signal in the Agrobacterium tumefaciens ADP‐glucose pyrophosphorylase
Source: Protein Sci. 2023 Sep 1;32(9):e4747. doi: 10.1002/pro.4747 (PMC10461462; doi:10.1002/pro.4747)
Supplement: Supplementary file 1 — Figure S1. Activator saturation curves of A. tumefaciens ADP‐Glc PPase mutants D141A and D141N at 4 mM of ATP. The effect of Pyr and Fru6P were assayed on the D141A and D141N mutant ADP‐Glc PPases. The assays were performed as described in Materials and Methods in the presence of 4 mM ATP. Figure S2. Binding analysis for the A. tumefaciens ADP‐Glc PPase WT and R11D. Panel A and B are for the wild‐type enzyme, and panel C and D for the mutant R11D. Thermals shifts of the melting points (T m ) at different concentration of activators Fru6P and Pyr have been performed as indicated in Material and Methods. Fitting of the data to obtain dissociation constants (K d) has also been described in Material and Methods. Figure S3. Sequence alignment for several species of plants and bacteria used for phylogenetic analysis. Highlighted are the homologous positions to Arg11 (region 1) and Asp141 (region 2) in Agrobacterium tumefaciens. Le column represents the code used in the phylogenetic tree (Figure S9). Figure S4. Sequence alignment for several species of photosynthetic eukaryotic ADP‐glucose pyrophosphorylase subunits. Highlighted are the homologous positions to Arg11 and Asp141 in Agrobacterium tumefaciens. Figure S5. Sequences of ADP‐glucose pyrophosphorylases used for phylogenetic analysis. Sequences, GI numbers, accession codes, and taxonomy were obtained as indicated in Materials and Methods. Figure S6. Sequences of ADP‐glucose pyrophosphorylases from plants used to analyze the conservancy of residues. Sequences, GI numbers, and accession codes were obtained as indicated in Materials and Methods. Figure S7. Dimer interaction between D148 and R14 in the E. coli ADP‐Glc PPase. Residues D148 and L18 are in homologous position to D141 and R11 in A. tumefaciens ADP‐Glc PPase, respectively. Figure has been constructed using the coordinates from the E. coli ADP‐Glc PPase structure (PDB: 5L6S) as indicated in Materials and Methods. Figure S8. Modeling of the N‐terminal domain i [file PRO-32-e4747-s001.pdf]

Supplementary material

(Figures)

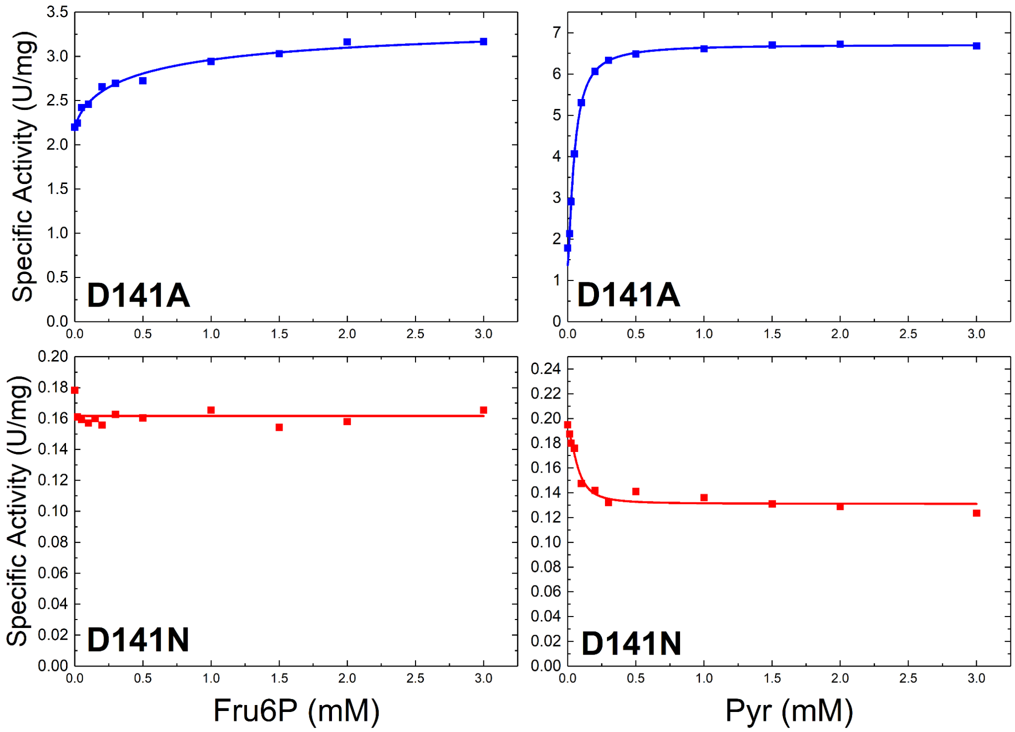

Enzyme	Fru6P $A_{0.5}$ (mM)	Fru6P V_{max} (U/mg)	Fru6P n_H	Fru6P Activation (V_m/V_0) (-fold)	Pyruvate $A_{0.5}$ (mM)	Pyruvate V_{max} (U/mg)	Pyruvate n_H	Pyruvate Activation (V_m/V_0) (-fold)
**D141A**	0.50 ± 0.32	3.45 ± 0.38	0.7 ± 0.3	1.6	0.049 ± 0.008	6.71 ± 0.09	1.4 ± 0.22	4.9
**D141N**	N/A	0.162 ± 0.003	N/A	1.0	0.075 ± 0.019[a]	0.131 ± 0.003	1.9 ± 0.72	0.69

[a] Parameter reflects inhibition ($I_{0.5}$) rather than activation ($A_{0.5}$)

Fig. S1. Activator saturation curves of A. tumefaciens ADP-Glc PPase mutants D141A and D141N at 4 mM of ATP. The effect of Pyr and Fru6P were assayed on the D141A and D141N mutant ADP-Glc PPases. The assays were performed as described in Materials and Methods in the presence of 4 mM ATP.

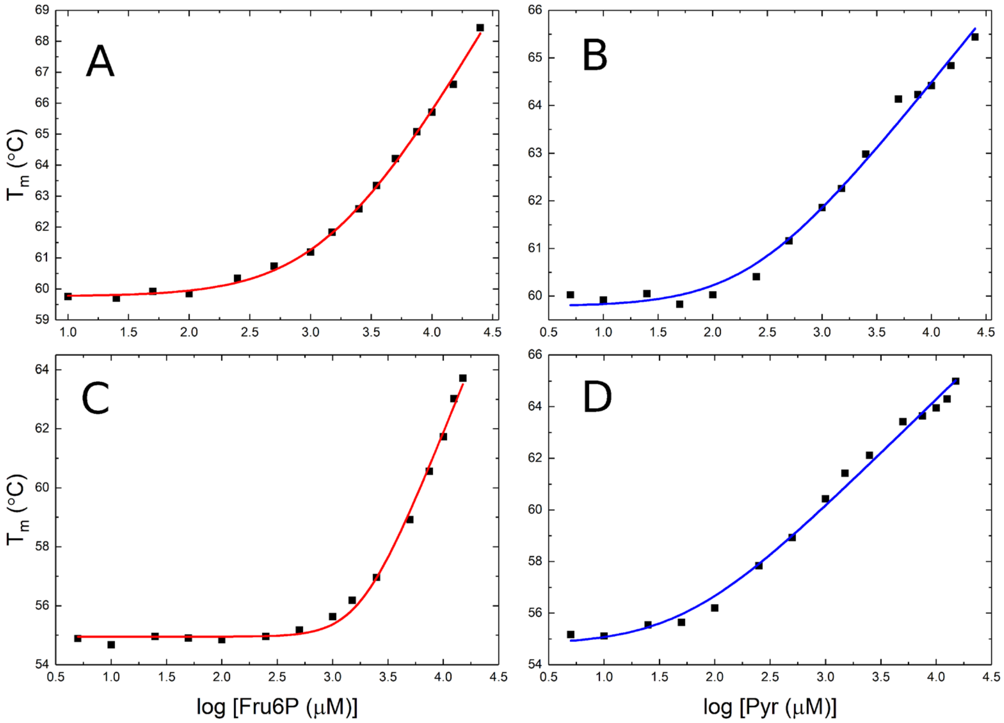

Fig. S2. Binding analysis for the *A. tumefaciens* ADP-Glc PPase WT and R11D.
Panel A and B are for the wild-type enzyme, and panel C and D for the mutant R11D. Thermals shifts of the melting points (T_m) at different concentration of activators Fru6P and Pyr have been performed as indicated in Material and Methods. Fitting of the data to obtain dissociation constants (K_d) has also been described in Material and Methods.

Fig S3. Sequence alignment for several species of plants and bacteria used for phylogenetic analysis. Highlighted are the homologous positions to Arg11 (region 1) and Asp141 (region 2) in *Agrobacterium tumefaciens*. Left column represents the code used in the phylogenetic tree (Fig. S9).

ID	Species	Region 1	Region 2
1_Atu	*Agrobacterium fabrum str. C58*	------MSEKRVQPLA**R**DAMAYVL	LAGDHIYKM**D**YEYMLQ
2_Rle	*Rhizobium leguminosarum*	------MVEKRVQPLA**R**DAMAYVL	LAGDHVYKM**D**YEWMLQ
3_Sfr	*Sinorhizobium fredii*	------MVEKRTQPLA**R**DAMAYVL	LAGDHIYKM**D**YELMLQ
4_Mam	*Mesorhizobium amorphae*	-----MADTKRTQPLA**R**DAMAYVL	LAGDHIYKM**D**YELMLR
5_Hph	*Hoeflea phototrophica*	------MEQKRAQPLA**R**DAMAYVL	LAGDHIYKM**D**YELMLQ
6_Ssa	*Sphingomonas sanguinis*	------MDLRRGQPLA**R**DAMAYVL	LAGDHVYKM**D**YELMLQ
7_Dch	*Devosia chinhatensis*	-----MVDQRVPSSLA**R**EAMAYVL	LAGDHIYKM**D**YEIMLR
8_Gbe	*Granulibacter bethesdensis*	--------MSGTSSIA**R**STMAYVL	LAGDHIYKM**D**YEIMLH
9_Rgi	*Roseomonas gilardii*	----MPLRPHAGSPLA**R**QAMAYVL	LAGDHVYKM**D**YEKMLR
10_Bdi	*Bradyrhizobium diazoefficiens USDA 110*	------MSAVGNEPLA**R**QALAFVL	LAGDHIYKM**D**YEVMLR
11_Rpa	*Rhodopseudomonas palustris BisA53*	------MTTGIEPPFA**R**HAMAYVL	LAGDHIYKM**D**YEKMLQ
12_Rud	*Rhodomicrobium udaipurense*	------MRPVGSSPLA**R**HAMAYVL	LAGDHVYKM**D**YEFMLQ
13_Rca	*Rhodobacter capsulatus SB 1003*	------MKPQPNQRLS**S**QAMAFVL	LAGDHIYKM**D**YEVMLL
14_Tin	*Thioclava indica*	------MLPQPNTRLS**A**QAMAFVL	LAGDHIYKM**D**YEIMLR
15_Rsp	*Rhodobacter sphaeroides ATCC 17025*	------MKAQPPLRLT**S**QAMAFVL	LAGDHVYKM**D**YEIMLR
16_Pde	*Paracoccus denitrificans*	-------MVQREERLS**R**RSMAFIL	LAGDHIYKM**D**YELMIR
17_Rsu	*Rhodovulum sulfidophilum*	-------METRYSRLA**S**RSMAFVL	LAGDHIYKM**D**YEIMLR
18_Ogr	*Oceanicola granulosus*	-----------MERLT**Q**RSMAFVL	LAGDHIYKM**D**YEIMLR
19_Ogu	*Oceaniovalibus guishaninsula*	------MTDRPNARLS**S**RTVAFVL	LAGDHIYKM**D**YEVMLA
20_Ili	*Inquilinus limosus*	-----MRDDGPIRQLP**K**RTIALVL	LAGDHVYKM**D**YAAMIE
21_Ali	*Azospirillum lipoferum*	-----MLDKRDLRLAP**R**RAVALVL	LAGDHIYKM**D**YGALLL
22_Aha	*Azospirillum halopraeferens*	---MDGLDKRDLRLAP**R**RAVALVL	LAGDHIYKM**D**YGALLL
23_Rru	*Rhodospirillum rubrum ATCC 11170*	-MDQITEFQLDINRAL**K**ETLALVL	LAGDHIYKQ**D**YSKLLA
24_Mma	*Magnetococcus marinus*	MADKSKNGVANLNEAI**R**QSLILVL	LGGDHIYKQ**D**YSVMLD
25_Rfe	*Rhodoferax ferrireducens T118*	------MKDGAHHRPV**R**RTISLVL	LAGDHVYKM**N**YASLIE
26_Pna	*Polaromonas naphthalenivorans CJ2*	---MKTNQLAQGLDLP**K**RAIALVL	LAGDHIYKM**N**YALMLA
27_Rge	*Rubrivivax gelatinosus IL144*	---MKPIDPASSFDLP**R**RSIALVL	LAGDHVYKQ**N**YALMLA
28_CAc	*Candidatus Accumulibacter phosphatis clade IIA str. UW-1*	------MVDRKTFQLP**R**KAIALVL	LAGDHVYKM**N**YAVMLV
29_Lmi	*Lautropia mirabilis ATCC 51599*	--MDNNARIDLERRLP**K**RAMALIL	LAGDHIYKM**D**YARMLA
30_Lch	*Leptothrix cholodnii SP-6*	---MDITRIAQARSLT**R**RSLALVL	LAGDHIYKM**D**YSIMLA
31_Tsp	*Thauera sp. MZ1T*	---MPAEAANRRRILT**R**RTLALVL	LAGDHVYKM**D**YSIMLE
32_Vpa	*Variovorax paradoxus S110*	SSNSTPQAPLQAHQLV**R**RTIALVL	LAGDHIYKM**D**YSIMVK
33_Bba	*Burkholderiales bacterium JOSHI_001*	-----MAAPMQSTQLV**R**RTIALVL	LAGDHIYKM**D**YSVMLK
34_Tin	*Thiomonas intermedia K12*	SPMNMQDRQMQPHMLV**R**RSIALVL	LAGDHVYKM**D**YSIMLQ

35_Bxe	*Burkholderia xenovorans LB400*	-----MDTPARLNDLQ**R**TTLAIVL	LAGDHIYKM**D**YTRMIA
36_Bph	*Burkholderia phymatum STM815*	-----MDTPASLNDLQ**H**TTLAIVL	LAGDHIYKM**D**YTRMVM
37_Ofo	*Oxalobacter formigenes OXCC13*	--------MLVASQLP**K**RTVALVL	LAGDHIYKM**D**YSMLLL
38_Smu	*Simonsiella muelleri ATCC 29453*	MNTPQTDNFLHNTDIA**K**DTLVLIL	LAGDHIYKM**D**YMNMVR
39_Nsp	*Nitrosomonas sp. Is79A3*	SQAECTTSTRFHNSVT**Y**NTLALIL	LGGDHIYKM**D**YSKLLD
40_Nsp	*Nitrosomonas sp. AL212*	SQAEYKSSSRFHSNIS**H**ETLALIL	LGGDHIYKM**D**YSKLLA
41_Sde	*Sulfuricella denitrificans skB26*	------EYPRFVSLLT**K**NTVALIL	LAGDHVYKM**D**YGEMLA
42_Sde	*Sulfuricella denitrificans skB26*	CPYRAELAPRFIGALT**K**KTYAMVL	LCGDHVYKM**D**YSRILA
43_Sde	*Sulfuricella denitrificans skB26*	----------------**M**KVLAMVL	FGADHIYRM**D**VRQMID
44_Mfl	*Methylobacillus flagellatus KT*	----ELSSSRFVSTLT**K**NTVALIL	LAGDHVYKM**D**YGQMLA
45_Mgl	*Methylovorus glucosetrophus SIP3-4*	----DLTSTRFISALT**K**NTVALIL	LAGDHIYKM**D**YGQMLA
46_Mmo	*Methylotenera mobilis JLW8*	-RQADQNSERFISTLT**K**NTVAMIL	LAGDHIYKM**D**YGKMLA
47_Mve	*Methylotenera versatilis 301*	----DQHSDRFISTLT**K**NTAAIIL	LAGDHIYKM**D**YGKMLA
48_Sli	*Sideroxydans lithotrophicus ES-1*	----EMRSPRFISALT**K**NTVALVL	LAGDHVYKM**D**YGEMLA
49_Gca	*Gallionella capsiferriformans ES-2*	--RCASSARPSVDQLT**K**NTYAMVL	LSGDHVYKM**D**YGKLLA
50_Gca	*Gallionella capsiferriformans*	HAASGDPEPRFVSRLT**K**NTYAMVL	LAGDHIYKM**D**YGKLLA
51_Dar	*Dechloromonas aromatica RCB*	CEMREQTDMRFISHLT**R**NTFAIIL	VAGDHIYKM**D**YGRMLA
52_Nmu	*Nitrosospira multiformis ATCC 25196*	--MKLPADLRSYSQIT**R**NSIAMIL	LGGDHVYKM**D**YAKLLA
53_Neu	*Nitrosomonas europaea ATCC 19718*	PAVQTNDNPRFVSTLT**R**NTLALIL	LGGDHIYKM**D**YGRILA
54_Neu	*Nitrosomonas eutropha C91*	----MNNNPRFVSTLT**R**NTLALIL	LGGDHIYKM**D**YGQILA
55_Tde	*Thiobacillus denitrificans ATCC 25259*	-----------------------M	FGADHIYRM**D**VRQMVR
56_Tde	*Thiobacillus denitrificans ATCC 25259*	RLYRYYTEPTLVTELT**R**KTLALVL	LGGDHVYTM**D**YTQMLL
57_Aar	*Aromatoleum aromaticum EbN1*	------------MIAG**K**SVLAFVM	FGADHVYRM**D**LRQMIE
58_Asp	*Azoarcus sp. BH72*	------------MIAG**K**SVLAFVM	FGADHVYRM**D**IRQMVQ
59_Aci	*Acidovorax citrulli AAC00-1*	------------MSST**K**NVLAIVM	FGADHIYRM**D**VRQMID
60_Asp	*Acidovorax sp. CF316*	------------MSRT**R**NVLAIVM	FGADHIYRM**D**VRQMVD
61_Nsp	*Neisseria sp. oral taxon 014 str. F0314*	-MNNNLSQVSSKYTLA**K**DTLVLIL	LAGDHIYKQ**D**YGQMLL
62_Ppe	*Proteus penneri ATCC 35198*	MTTEQGQKLMLAQQLP**K**EAIALVL	LAGDHIYKM**N**YARLLL
63_Eco	*Escherichia coli str. K-12 substr. MG1655*	VSLEKNDHLMLARQLP**L**KSVALIL	LAGDHIYKQ**D**YSRMLI
64_Ype	*Yersinia pestis A1122*	VRFESTDSLMLARQLP**N**KTVALIL	LAGDHIYKM**D**YSRMLL
65_Pal	*Providencia alcalifaciens DSM 30120*	SYPNSIPRQWLAKELP**N**NTVALIL	LAGDHIYKM**D**YSRMLL
66_Asp	*Alteromonas sp. SN2*	-----MNAEIDLQQII**E**NTMVLVL	LSGDHVYQM**D**YRKLLA
67_Asp	*Alteromonas sp. SN2*	----------YISNLT**R**DTYALIL	LSGDHIYRM**D**YGTMLA
68_Cho	*Cardiobacterium hominis ATCC 15826*	-MTPKENPLPSTEEMA**N**ETLVLIL	LAGDHIYKM**D**YMQMIR
69_Pda	*Pasteurella dagmatis ATCC 43325*	IMNSSIAKLPNKYELV**K**DTLVLIL	LAGDHIYKQ**D**YSQMLL
70_Aca	*Acidithiobacillus caldus ATCC 51756*	GITGVANSPRFVSNLT**K**NSLALVL	LAGDHIYRM**D**YGQMLA
71_Pin	*Psychromonas ingrahamii 37*	---------------M**A**KVLSMIL	FGSDHIYKM**D**IRQKIA

72_Pin	*Psychromonas ingrahamii 37*	---------------M**A**GILAMIL	FGSDHIYKM**D**VQQMVE
73_Pin	*Psychromonas ingrahamii 37*	----------YISNLT**K**DTYALVL	LSGDHVYRM**D**YGPLIA
74_Msp	*Moritella sp. PE36*	---------------M**Q**DTLTVIL	LSGDHIYRM**D**YAPMLE
75_Msp	*Moritella sp. PE36*	----------YISNLT**K**DTYALIL	LSGDHVYRM**D**YGDLLA
76_Aba	*Alteromonadales bacterium TW-7*	----------YISSLT**R**ETYALIL	LSGDHVYRM**D**YGALLA
77_Nmo	*Nitrococcus mobilis Nb-231*	---------RFVSRLT**R**DTLAIIL	LAGDHVYKM**D**YGPMIA
78_Nmo	*Nitrococcus mobilis Nb-231*	----------FVSRLT**R**DTLALVL	LSGDHIYKM**D**YGALLA
79_Cja	*Cellvibrio japonicus Ueda107*	--------GRFVSRLT**R**ETLAVIL	LAGDHIYKM**D**YGTMIA
80_Nha	*Nitrosococcus halophilus Nc 4*	---------RFVSRLT**R**DTLALIL	LAGDHVYKM**D**YGDMLA
81_Nha	*Nitrosococcus halophilus Nc 4*	---------MRKKRYG**G**RIIAFVM	FGADHVYRM**D**VRQMAW
82_Nwa	*Nitrosococcus watsonii C-113*	---------RFVSRLT**R**DTLALIL	LAGDHVYKM**D**YGDMLA
83_Mtu	*Methylobacter tundripaludum SV96*	------KHDHNISHLT**R**NTIALIL	LAGDHIYKM**D**YGAMLA
84_Mtu	*Methylobacter tundripaludum SV96*	--------------ML**D**KTLTIIL	LSGDHIYRM**D**YAAMLQ
85_Aeh	*Alkalilimnicola ehrlichii MLHE-1*	---------RFVSRLT**R**DTLALIM	LAGDHVYKM**D**YGGMIA
86_Aeh	*Alkalilimnicola ehrlichii MLHE-1*	---------RFVSRLT**R**NTLVLIL	LAGDHVYKM**D**YGPMLA
87_Hha	*Halorhodospira halophila SL1*	---------RFVSRLT**R**DTLALIL	LAGDHIYKM**D**YGPLLA
88_Hha	*Halorhodospira halophila SL1*	---------RFVSRLT**R**ETLALIM	LAGDHVYKM**D**YGAMIA
89_Hef	*Hydrocarboniphaga effusa AP103*	---------RFVSRLT**K**STLALVM	LAGDHVYKM**D**YGRMLA
90_Tth	*Thioalkalivibrio thiocyanoxidans ARh 4*	-------------MQT**D**RIIAFVM	FGADHIYRM**D**IRQMVA
91_Tth	*Thioalkalivibrio thiocyanoxidans ARh 4*	--------QRFVSRLT**R**ETLALIL	LAGDHIYKM**D**YGQMIA
92_Vba	*Vibrionales bacterium SWAT-3*	----MHNHKYIEVLTM**Q**DTLTIVL	LSGDHIYRM**D**YAPMLK
93_Vba	*Vibrionales bacterium SWAT-3*	---------------M**A**GVLGMIL	FGSDHIYKM**D**IKQMLD
94_Vch	*Vibrio cholerae 12129(1)*	---------------M**Q**DTLAVIL	LSGDHIYRM**D**YAAMLE
95_Vch	*Vibrio cholerae 12129(1)*	---------------M**A**GVLGMIL	FGSDHIYKM**D**IRQMLD
96_Dac	*Desulfuromonas acetoxidans DSM 684*	-----------VSELT**R**NTLALVL	LGGDHIYAM**D**YRDMIA
97_Dac	*Desulfobacca acetoxidans DSM 11109*	------------MDRL**R**KLTTLIM	LSGDHVYKM**N**YMEMLN
98_Dti	*Desulfomonile tiedjei DSM 6799*	---------MNLRSEM**K**KTLCILM	LSGDHVYKM**N**YNDMLA
99_Dau	*Desulfobacterium autotrophicum HRM2*	--------------MQ**N**QTLTFLL	LSGDHIYRM**D**YAAMLS
100_Dau	*Desulfobacterium autotrophicum HRM2*	--------MEITAMIM**K**DVLGLIM	LAGDHLYRM**D**YRKFVQ
101_Dau	*Desulfobacterium autotrophicum HRM2*	---------------M**G**GILSMIL	FGGDHIYKM**E**IRQMID
102_Gme	*Geobacter metallireducens GS-15*	--------MYVTGNLA**G**NTIAMVL	FGGDHIYRM**D**VSQMIG
103_Sce	*Sorangium cellulosum So ce56*	---------------------MIL	FGGDHVYKM**D**VRQMLH
104_Hoc	*Haliangium ochraceum DSM 14365*	--MEGDYAQHIHVRPQ**P**RVLAVVL	FGGDHIYRM**D**VRQMLS
105_Mtu	*Mycobacterium tuberculosis T85*	------------MREV**P**HVLGIVL	FGADHVYRM**D**PEQMVR
106_Sco	*Streptomyces coelicolor A3(2)*	-----------MRRGG**P**SVLGIVL	FGADHVYRM**D**PRQMLA
107_Str	*Streptomyces*	-----------MRRGG**P**SVLGIVL	FGADHVYRM**D**PRQMLA
108_CMe	*Candidatus Melainabacteria bacterium MEL.A1*	-------------MSI**D**DALVMIL	FGGDHIYKM**D**VSQMLD

109_CMe	*Candidatus Melainabacteria bacterium GWF2_37_15*	-------------MYKDDVMVMIM	FGGDHIYKMDVKHMLK
110_Vin	*Vulgatibacter incomptus*	---------------MSRTLALIL	FGADHVYRMDVRQMLH
111_Cll	*Corallococcus llansteffanensis*	---------------MSKLLAMIL	FGADHVYRMDVRKMLD
112_Sce	*Sorangium cellulosum*	-------------MKHNDVVVLIL	LSGDHLYRMDYEPFLE
113_Sno	*Selenomonas noxia ATCC 43541*	-------------MKKTECLAMIL	LSGDHIYTMDYAWMLE
114_Bsu	*Bacillus subtilis subsp. spizizenii ATCC 6633*	--------------MKKQCVAMLL	LSGDHIYKMDYGKMLD
115_Mau	*Mahella australiensis 50-1 BON*	-------------MRKREVVAMIL	LSGDHIYKMNYNKMIA
116_Lac	*Lactobacillus acidophilus 30SC*	--------------MSTKMLGLIL	LSGDHIYKMDYEDMLE
117_Smu	*Streptococcus mutans GS-5*	--------------MKNEMLALIL	LSGDHIYKMDYDDMLQ
118_Aar	*Acetohalobium arabaticum DSM 5501*	----------------METLAMVL	LSSDHVYKMDYSKMVN
119_Rce	*Ruminiclostridium cellulolyticum*	-------------MIRKEMIAMLL	LSGDHIYKMDYSKMLD
120_Tbr	*Treponema brennaborense DSM 12168*	---------------MAKVLSIIL	LSGDQLYRMDLKKFLD
121_Saf	*Spirochaeta africana DSM 8902*	-------MRRRVDPGSNDVLSIIL	LSGDQLYRMDLAEMYR
122_Sco	*Sphaerochaeta coccoides DSM 17374*	------------MKQKNRAIAIVL	LSGDQLYRMDFQLMLK
123_Bhy	*Brachyspira hyodysenteriae WA1*	-------------MRAFNTVALIL	LSGDQVYRMNYNVMLQ
124_Nsp	*Nostoc sp. PCC 7120*	---------------MKKVLAIIL	LSGDHLYRMDYRLFIQ
125_Ama	*Arthrospira maxima CS-328*	---------MYQVIEVKQVLAVIL	LSGDHLYRMDYRDFVQ
126_Ssp	*Synechocystis sp. PCC 6803*	-----MCCWQSRGLLVKRVLAIIL	LSGDHLYRMDYAQFVK
127_Pma	*Prochlorococcus marinus str. AS9601*	---------------MKRVLAIIL	LSGDQLYRMDYSLFVQ
128_Gvi	*Gloeobacter violaceus PCC 7421*	---------------MRQVTAIIL	LSGDHLYRMDYSKFVR
129_CPr	*Candidatus Protochlamydia amoebophila UWE25*	RSSQVVETSDSQSVDMRQVASLIL	LSGDQLYNIDFQEMVH
130_Pac	*Parachlamydia acanthamoebae UV-7*	KTTPLTQTINLHTHRTDRVASIIL	LSGDQLYNMDFRPMLQ
131_Cpe	*Chlamydophila pecorum E58*	GFQNHLSGGKTSYRYRDRVGVIVL	LSGDQLYNMDFHVIVE
132_Cpn	*Chlamydophila pneumoniae AR39*	DFP-EASNFESSHFYRDKVGVIIL	LSGDQLYNMDFRSIVD
133_Cfe	*Chlamydophila felis Fe/C-56*	DFQGYPPNYQASHFYRDKVGVIVL	LSGDQLYNMDFRKIVD
134_Ctr	*Chlamydia trachomatis L2tet1*	RTKEEQINRKRSHFYRDNVGVIVL	LSGDQLYNMDFRSIVD
135_StuS	*Solanum tuberosum (Small Subunit)*	VSDSQNSQTCLDPDASRSVLGIIL	LAGDHLYRMDYEKFIQ
136_AthS	*Arabidopsis thaliana (Small subunit)*	VSDSQNSQTCLDPDASSSVLGIIL	LAGDHLYRMDYEKFIQ
137_OtaS	*Ostreococcus tauri (Small subunit)*	---------------MDNVLSIIL	LSGDHLYRMDYRPFIM
138_OtaL	*Ostreococcus tauri (Large subunit)*	-----------AIANTKTVAAVIL	LAGDHLYRMDYMKFVE
139_AthL1	*Arabidopsis thaliana (APL1)*	AGESKVQELETEKRDPRTVASIIL	LSGDHLYRMDYMDFIQ
140_AthL2	*Arabidopsis thaliana (APL2)*	---SHEPLLKTQNADPKNVASIIL	LSGDHLYRMDYMNFVQ
141_ZmaSh2	*Zea mays (Sh2)*	TLHSQTQSSRKNYADANRVSAIIL	LSGDQLYRMNYMELVQ
142_StuL	*Solanum tuberosum (Large Subunit)*	TVFVDMPRLERRRANPKDVAAVIL	LSGDHLYRMDYMELVQ
143_AthL3	*Arabidopsis thaliana (APL3)*	ALKNQPSMFERRRADPKNVAAIIL	LSGDHLYRMNYMDFVQ
144_AthL4	*Arabidopsis thaliana (APL4)*	AMTVKTSMFERRKVDPQNVAAIIL	LSGDHLYRMNYMDFVQ

Fig. S4. Sequence alignment for several species of photosynthetic eukaryotic ADP-glucose pyrophosphorylase subunits. Highlighted are the homologous positions to Arg11 and Asp141 in *Agrobacterium tumefaciens*.

#	Species	Region 1	Region 2
**1**	*Arabidopsis lyrata subsp. lyrata*	----ELETEKRDS**R**TVASIILGGGAGT	LILSGDHLYRM**D**YMDFVQDHR
**2**	*Arabidopsis lyrata subsp. lyrata*	----MFERRKADP**Q**NVAAIILGGGNGA	LILSGDHLYRM**N**YMDFVQSHV
**3**	*Arabidopsis thaliana*	----ELETEKRDP**R**TVASIILGGGAGT	LILSGDHLYRM**D**YMDFIQDHR
**4**	*Arabidopsis thaliana*	----MFERRRADP**K**NVAAIILGGGDGA	IILSGDHLYRM**N**YMDFVQHHV
**5**	*Arabidopsis thaliana*	----MFERRKVDP**Q**NVAAIILGGGNGA	LILSGDHLYRM**N**YMDFVQSHV
**6**	*Arabidopsis thaliana*	----LLKTQNADP**K**NVASIILGGGAGT	LILSGDHLYRM**D**YMNFVQKHI
**7**	*Arabidopsis thaliana*	----QTCLDPDAS**S**SVLGIILGGGAGT	LILAGDHLYRM**D**YEKFIQAHR
**8**	*Arabidopsis thaliana*	----QTCLDPDAS**S**SVLGIILGGGAGT	LILAGDHLYRM**D**YEKFIQAHR
**9**	*Brachypodium distachyon*	----DADTETRNA**R**TVVAVILGGGAGT	IILSGDHLYRM**D**YMDFVQSHR
**10**	*Brachypodium distachyon*	-----FRRNYADP**N**EVAAVILGGGTGT	LILSGDQLYRM**D**YMELVQKHV
**11**	*Brachypodium distachyon*	----QTCLDPDAS**T**SVLGIILGGGAGT	LILAGDHLYRM**D**YEKFIQAHR
**12**	*Chlamydomonas reinhardtii*	----QNDPAGDIS**K**TVLGIILGGGAGT	LILSGDHLYRM**D**YRDFVRKHR
**13**	*Glycine max*	----SFLRRRADP**K**NVISIILGGGPGT	LILAGDHLYRM**D**YMDLVQSHV
**14**	*Glycine max*	----TFEKPEVDP**K**SVASIILGGGAGT	LILSGDHLYRM**D**YMNFVQRHV
**15**	*Glycine max*	----SFLRRRADP**K**NVVSIILGGGPGT	LILAGDHLYRM**D**YMDLVQSHV
**16**	*Glycine max*	----TFEKPEVDP**K**SVASIILGGGAGT	LILSGDHLYRM**D**YMDFVQRHV
**17**	*Glycine max*	----SFLRRKADP**K**NVVSVILGGGPGI	LILAGDHLYRM**N**YMDLVQSHV
**18**	*Glycine max*	----SFLRRKADP**K**NVVSIILGGGPGI	LILAGDHLYRM**D**YMDLVQSHV
**19**	*Glycine max*	----IFQSPKANP**E**NVVAIILGGGAGT	LIISGDHLCRM**D**YMKLVEKHI
**20**	*Glycine max*	----IFQNPKANP**E**NVAAIILGGGAGT	LIISGDHLCRM**D**YMKLLEKHI
**21**	*Glycine max*	----DLDMERRNP**R**TVLAVILGGGAGT	LILSGDHLYRM**D**YMDFVQNHR
**22**	*Medicago truncatula*	----QTCLDPDAS**R**SVLGIILGGGAGT	LVLAGDHLYRM**D**YERFIQAHR
**23**	*Medicago truncatula*	----SFIRRKADP**K**NVASIVLGGGPGV	IILAGDHLYRM**D**YMDLVQSHI
**24**	*Medicago truncatula*	----QTCLDPDAS**R**SVLGIILGGGAGT	LILAGDHLYRM**D**YEKFIQAHR
**25**	*Micromonas commoda*	-----AQSAISNS**K**SVAAVILGGGAGT	VILSGDHLYRM**D**YMAFVARHR
**26**	*Micromonas commoda*	------TEVNDNT**D**NVLGIILGGGAGT	IILSGDHLYRM**D**YKPFILKHR
**27**	*Micromonas pusilla CCMP1545*	-----GKVVDDST**D**NVLAIILGGGAGT	IILSGDHLYRM**D**YKPFILEHR
**28**	*Micromonas pusilla CCMP1545*	-----TSTVISNS**K**SVAAVILGGGAGT	VILSGDHLYRM**D**YMAFVDRHR
**29**	*Ostreococcus lucimarinus CCE9901*	-----STTAIANS**K**TVAAVILGGGAGT	VILAGDHLYRM**D**YMKFVEAHR
**30**	*Ostreococcus tauri*	-----SPNAIANT**K**TVAAVILGGGAGT	VILAGDHLYRM**D**YMKFVEAHR
**31**	*Ostreococcus tauri*	-----AAAANDGM**D**NVLSIILGGGAGT	LILSGDHLYRM**D**YRPFIMKHR
**32**	*Ostreococus lucimarinus CCE9901*	------------M**D**NVLSIILGGGAGT	LILSGDHLYRM**D**YRPFIRDHR
**33**	*Populus trichocarpa*	----VFETPQADP**S**NVASIILGGGAGT	LILSGDHLYRM**N**YMEFVQKHI

**34**	*Populus trichocarpa*	----DLEMEKRDP**R**TVVAVILGGGAGT	LILSGDHLYRM**D**YMDFVQNHR
**35**	*Populus trichocarpa*	----RFERRKADP**K**NVASIILGGGAGT	LVLSGDHLYRM**D**YMDFVQHHI
**36**	*Ricinus communis*	----VFETPRADP**K**KVASIILGGGAGT	LILSGDHLYRM**D**YMEFVQKHI
**37**	*Ricinus communis*	PPPPRFERRKVDP**K**NVASIILGGGAGT	LILSGDHLYRM**D**YMDFVQHHV
**38**	*Ricinus communis*	----DLEMEKRDP**R**TVVAIILGGGAGT	LVLSGDHLYRM**D**YMDFVQNHR
**39**	*Selaginella moellendorffii*	---SVFETPRVDP**K**SVVSIILGGGVGT	LVLSGDHLYRM**D**YMDFVQKHK
**40**	*Solanum tuberosum*	----QTCLDPDAS**R**SVLGIILGGGAGT	LILAGDHLYRM**D**YEKFIQAHR
**41**	*Solanum tuberosum*	----RLERRRANP**K**DVAAVILGGGEGT	VVLSGDHLYRM**D**YMELVQNHI
**42**	*Sorghum bicolor*	----DQALEARNS**R**TVVAVILGGGAGT	LILSGDHLYRM**D**YMDFVQSHR
**43**	*Sorghum bicolor*	----QTCLDPDAS**T**SVLGIILGGGAGT	LILAGDHLYRM**D**YQKFIQAHR
**44**	*Sorghum bicolor*	-----SRKSYADA**N**HVSAIILGGGTGS	VILSGDQLYQM**N**YMELVQKHV
**45**	*Sorghum bicolor*	-----FRRNYADP**N**EVAAVILGGGTGT	LILSGDQLYRM**D**YMELVQKHV
**46**	*Vitis vinifera*	----DLEMEKRDP**R**TVVAVILGGGAGT	LILSGDHLYRM**D**YMDFVQNHR
**47**	*Vitis vinifera*	----QTCLDPDAS**R**SVLGIILGGGAGT	LVLAGDHLYRM**D**YERFIQAHR
**48**	*Vitis vinifera*	----VFEKQHADP**S**SVAAIILGGGAGT	LILSGDHLYRM**D**YMDFVQKHI
**49**	*Vitis vinifera*	----IFERRRADP**K**NVASIILGGGAGT	LILSGDHLYRM**D**YMDLVQNHI
**50**	*Volvox carteri f. nagariensis*	-----EPATKART**N**TVLSIILGGGAGT	LILSGDHLYRM**D**YMKFVNYHR
**51**	*Volvox carteri f. nagariensis*	----SYDYAGDIS**K**TVLGIILGGGAGT	LILSGDHLYRM**D**YRDFVRKHR
**52**	*Zea mays*	-----SRKNYADA**N**RVSAIILGGGTGS	VILSGDQLYRM**N**YMELVQKHV
**53**	*Zea mays*	----DQALEARNS**K**TVVAVILGGGAGT	LILSGDHLYRM**D**YMDFVQSHR
**54**	*Zea mays*	-----TYLNPQAH**D**SVLGIILGGGAGT	LILAGDHLYRM**D**YEKFIQAHR
**55**	*Zea mays*	----QTCLDPDAS**T**SVLGIILGGGAGT	LILAGDHLYRM**D**YQKFIQAHR
**56**	*Zea mays*	------ARRDVSP**D**TVASIILGGGAGT	LILSGDHLYRM**D**YMDFVQKHV
**57**	*Zea mays*	-----SRKNYADA**N**RVSAIILGGGTGS	VILSGDQLYRM**N**YMELVQKHV

Fig. S5. Sequences of ADP-glucose pyrophosphorylases used for phylogenetic analysis. Sequences, GI numbers, accession codes, and taxonomy were obtained as indicated in Materials and Methods.

Code	GI number	Species	Accession	Taxon
1_Atu	15890896	*Agrobacterium fabrum str. C58*	NP_356568.1	Alphaproteobacteria
2_Rle	516587070	*Rhizobium leguminosarum*	WP_017962109.1	Alphaproteobacteria
3_Sfr	504095815	*Sinorhizobium fredii*	WP_014329809.1	Alphaproteobacteria
4_Mam	493229187	*Mesorhizobium amorphae*	WP_006206262.1	Alphaproteobacteria
5_Hph	750145252	*Hoeflea phototrophica*	WP_040449329.1	Alphaproteobacteria
6_Ssa	970452290	*Sphingomonas sanguinis*	WP_058732779.1	Alphaproteobacteria
7_Dch	805450903	*Devosia chinhatensis*	WP_046104745.1	Alphaproteobacteria
8_Gbe	644456638	*Granulibacter bethesdensis*	WP_025318349.1	Alphaproteobacteria
9_Rgi	653031067	*Roseomonas gilardii*	WP_027282834.1	Alphaproteobacteria
10_Bdi	27381569	*Bradyrhizobium diazoefficiens USDA 110*	NP_773098.1	Alphaproteobacteria
11_Rpa	115522826	*Rhodopseudomonas palustris BisA53*	YP_779737.1	Alphaproteobacteria
12_Rud	739376416	*Rhodomicrobium udaipurense*	WP_037237335.1	Alphaproteobacteria
13_Rca	294677377	*Rhodobacter capsulatus SB 1003*	YP_003577992.1	Alphaproteobacteria
14_Tin	740292445	*Thioclava indica*	WP_038131090.1	Alphaproteobacteria
15_Rsp	146277181	*Rhodobacter sphaeroides ATCC 17025*	YP_001167340.1	Alphaproteobacteria
16_Pde	500074640	*Paracoccus denitrificans*	WP_011750653.1	Alphaproteobacteria
17_Rsu	985595180	*Rhodovulum sulfidophilum*	WP_060834681.1	Alphaproteobacteria
18_Ogr	494465075	*Oceanicola granulosus*	WP_007254599.1	Alphaproteobacteria
19_Ogu	494667949	*Oceaniovalibus guishaninsula*	WP_007425892.1	Alphaproteobacteria
20_Ili	652474393	*Inquilinus limosus*	WP_026869087.1	Alphaproteobacteria
21_Ali	504012613	*Azospirillum lipoferum*	WP_014246607.1	Alphaproteobacteria
22_Aha	737724709	*Azospirillum halopraeferens*	WP_035693542.1	Alphaproteobacteria
23_Rru	83593581	*Rhodospirillum rubrum ATCC 11170*	YP_427333.1	Alphaproteobacteria
24_Mma	500032464	*Magnetococcus marinus*	WP_011713182.1	Alphaproteobacteria
25_Rfe	89899325	*Rhodoferax ferrireducens T118*	YP_521796.1	Betaproteobacteria
26_Pna	121604015	*Polaromonas naphthalenivorans CJ2*	YP_981344.1	Betaproteobacteria
27_Rge	383758760	*Rubrivivax gelatinosus IL144*	YP_005437745.1	Betaproteobacteria
28_CAc	257093709	*Candidatus Accumulibacter phosphatis clade IIA str. UW-1*	YP_003167350.1	Betaproteobacteria
29_Lmi	319944837	*Lautropia mirabilis ATCC 51599*	ZP_08019099.1	Betaproteobacteria
30_Lch	171058572	*Leptothrix cholodnii SP-6*	YP_001790921.1	Betaproteobacteria
31_Tsp	217970454	*Thauera sp. MZ1T*	YP_002355688.1	Betaproteobacteria
32_Vpa	239813432	*Variovorax paradoxus S110*	YP_002942342.1	Betaproteobacteria
33_Bba	375105315	*Burkholderiales bacterium JOSHI_001*	ZP_09751576.1	Betaproteobacteria
34_Tin	296135572	*Thiomonas intermedia K12*	YP_003642814.1	Betaproteobacteria
35_Bxe	91782889	*Burkholderia xenovorans LB400*	YP_558095.1	Betaproteobacteria
36_Bph	186476555	*Burkholderia phymatum STM815*	YP_001858025.1	Betaproteobacteria
37_Ofo	237748940	*Oxalobacter formigenes OXCC13*	ZP_04579420.1	Betaproteobacteria
38_Smu	294788710	*Simonsiella muelleri ATCC 29453*	ZP_06753951.1	Betaproteobacteria
39_Nsp	339484394	*Nitrosomonas sp. Is79A3*	YP_004696180.1	Betaproteobacteria
40_Nsp	325983317	*Nitrosomonas sp. AL212*	YP_004295719.1	Betaproteobacteria

41_Sde	394987804	*Sulfuricella denitrificans skB26*	ZP_10380643.1	Betaproteobacteria
42_Sde	394987872	*Sulfuricella denitrificans skB26*	ZP_10380711.1	Betaproteobacteria
43_Sde	394988308	*Sulfuricella denitrificans skB26*	ZP_10381146.1	Betaproteobacteria
44_Mfl	91775721	*Methylobacillus flagellatus KT*	YP_545477.1	Betaproteobacteria
45_Mgl	253998981	*Methylovorus glucosetrophus SIP3-4*	YP_003051044.1	Betaproteobacteria
46_Mmo	253996202	*Methylotenera mobilis JLW8*	YP_003048266.1	Betaproteobacteria
47_Mve	297538687	*Methylotenera versatilis 301*	YP_003674456.1	Betaproteobacteria
48_Sli	291614267	*Sideroxydans lithotrophicus ES-1*	YP_003524424.1	Betaproteobacteria
49_Gca	302877866	*Gallionella capsiferriformans ES-2*	YP_003846430.1	Betaproteobacteria
50_Gca	503058355	*Gallionella capsiferriformans*	WP_013293331.1	Betaproteobacteria
51_Dar	71906225	*Dechloromonas aromatica RCB*	YP_283812.1	Betaproteobacteria
52_Nmu	82701851	*Nitrosospira multiformis ATCC 25196*	YP_411417.1	Betaproteobacteria
53_Neu	30249970	*Nitrosomonas europaea ATCC 19718*	NP_842040.1	Betaproteobacteria
54_Neu	114331286	*Nitrosomonas eutropha C91*	YP_747508.1	Betaproteobacteria
55_Tde	74317193	*Thiobacillus denitrificans ATCC 25259*	YP_314933.1	Betaproteobacteria
56_Tde	74318079	*Thiobacillus denitrificans ATCC 25259*	YP_315819.1	Betaproteobacteria
57_Aar	56479382	*Aromatoleum aromaticum EbN1*	YP_160971.1	Betaproteobacteria
58_Asp	119898088	*Azoarcus sp. BH72*	YP_933301.1	Betaproteobacteria
59_Aci	120611645	*Acidovorax citrulli AAC00-1*	YP_971323.1	Betaproteobacteria
60_Asp	395008322	*Acidovorax sp. CF316*	ZP_10391995.1	Betaproteobacteria
61_Nsp	298369492	*Neisseria sp. oral taxon 014 str. F0314*	ZP_06980809.1	Betaproteobacteria
62_Ppe	226330079	*Proteus penneri ATCC 35198*	ZP_03805597.1	Gammaproteobacteria
63_Eco	16131304	*Escherichia coli str. K-12 substr. MG1655*	NP_417888.1	Gammaproteobacteria
64_Ype	384138208	*Yersinia pestis A1122*	YP_005520910.1	Gammaproteobacteria
65_Pal	212710453	*Providencia alcalifaciens DSM 30120*	ZP_03318581.1	Gammaproteobacteria
66_Asp	333892129	*Alteromonas sp. SN2*	YP_004466004.1	Gammaproteobacteria
67_Asp	333893518	*Alteromonas sp. SN2*	YP_004467393.1	Gammaproteobacteria
68_Cho	258543877	*Cardiobacterium hominis ATCC 15826*	ZP_05704111.1	Gammaproteobacteria
69_Pda	260914094	*Pasteurella dagmatis ATCC 43325*	ZP_05920567.1	Gammaproteobacteria
70_Aca	255020713	*Acidithiobacillus caldus ATCC 51756*	ZP_05292773.1	Gammaproteobacteria
71_Pin	119946655	*Psychromonas ingrahamii 37*	YP_944335.1	Gammaproteobacteria
72_Pin	119946654	*Psychromonas ingrahamii 37*	YP_944334.1	Gammaproteobacteria
73_Pin	119945042	*Psychromonas ingrahamii 37*	YP_942722.1	Gammaproteobacteria
74_Msp	149911154	*Moritella sp. PE36*	ZP_01899779.1	Gammaproteobacteria
75_Msp	149910892	*Moritella sp. PE36*	ZP_01899524.1	Gammaproteobacteria
76_Aba	119468777	*Alteromonadales bacterium TW-7*	ZP_01611829.1	Gammaproteobacteria
77_Nmo	88811612	*Nitrococcus mobilis Nb-231*	ZP_01126866.1	Gammaproteobacteria
78_Nmo	88813574	*Nitrococcus mobilis Nb-231*	ZP_01128807.1	Gammaproteobacteria
79_Cja	192360471	*Cellvibrio japonicus Ueda107*	YP_001982358.1	Gammaproteobacteria
80_Nha	292491218	*Nitrosococcus halophilus Nc 4*	YP_003526657.1	Gammaproteobacteria
81_Nha	292491590	*Nitrosococcus halophilus Nc 4*	YP_003527029.1	Gammaproteobacteria
82_Nwa	300113472	*Nitrosococcus watsonii C-113*	YP_003760047.1	Gammaproteobacteria
83_Mtu	344940183	*Methylobacter tundripaludum SV96*	ZP_08779471.1	Gammaproteobacteria
84_Mtu	344943819	*Methylobacter tundripaludum SV96*	ZP_08783105.1	Gammaproteobacteria

85_Aeh	114319816	*Alkalilimnicola ehrlichii MLHE-1*	YP_741499.1	Gammaproteobacteria
86_Aeh	114320119	*Alkalilimnicola ehrlichii MLHE-1*	YP_741802.1	Gammaproteobacteria
87_Hha	121997897	*Halorhodospira halophila SL1*	YP_001002684.1	Gammaproteobacteria
88_Hha	121998643	*Halorhodospira halophila SL1*	YP_001003430.1	Gammaproteobacteria
89_Hef	392953280	*Hydrocarboniphaga effusa AP103*	ZP_10318834.1	Gammaproteobacteria
90_Tth	350561659	*Thioalkalivibrio thiocyanoxidans ARh 4*	ZP_08930497.1	Gammaproteobacteria
91_Tth	350561821	*Thioalkalivibrio thiocyanoxidans ARh 4*	ZP_08930659.1	Gammaproteobacteria
92_Vba	148975147	*Vibrionales bacterium SWAT-3*	ZP_01812071.1	Gammaproteobacteria
93_Vba	148977548	*Vibrionales bacterium SWAT-3*	ZP_01814127.1	Gammaproteobacteria
94_Vch	229528475	*Vibrio cholerae 12129(1)*	ZP_04417866.1	Gammaproteobacteria
95_Vch	229529245	*Vibrio cholerae 12129(1)*	ZP_04418635.1	Gammaproteobacteria
96_Dac	95930373	*Desulfuromonas acetoxidans DSM 684*	ZP_01313110.1	Deltaproteobacteria
97_Dac	328953288	*Desulfobacca acetoxidans DSM 11109*	YP_004370622.1	Deltaproteobacteria
98_Dti	392410016	*Desulfomonile tiedjei DSM 6799*	YP_006446623.1	Deltaproteobacteria
99_Dau	224367273	*Desulfobacterium autotrophicum HRM2*	YP_002601436.1	Deltaproteobacteria
100_Dau	224367976	*Desulfobacterium autotrophicum HRM2*	YP_002602139.1	Deltaproteobacteria
101_Dau	224369989	*Desulfobacterium autotrophicum HRM2*	YP_002604153.1	Deltaproteobacteria
102_Gme	78223964	*Geobacter metallireducens GS-15*	YP_385711.1	Deltaproteobacteria
103_Sce	162455376	*Sorangium cellulosum So ce56*	YP_001617743.1	Deltaproteobacteria
104_Hoc	262198960	*Haliangium ochraceum DSM 14365*	YP_003270169.1	Deltaproteobacteria
105_Mtu	289757310	*Mycobacterium tuberculosis T85*	ZP_06516688.1	Actinobacteridae
106_Sco	161353683	*Streptomyces coelicolor A3(2)*	NP_625258.2	Actinomycetes
107_Str	499337764	*Streptomyces*	WP_011027472.1	Actinomycetes
108_CMe	1079475190	*Candidatus Melainabacteria bacterium MEL.A1*		Melainabacteria
109_CMe	1083715065	*Candidatus Melainabacteria bacterium GWF2_37_15*		Melainabacteria
110_Vin	914793558	*Vulgatibacter incomptus*	WP_050724205.1	Myxococcota
111_Cll	1488083595	*Corallococcus llansteffanensis*	WP_120645735.1	Myxococcota
112_Sce	501194959	*Sorangium cellulosum*	WP_012237977.1	Myxococcota
113_Sno	292670340	*Selenomonas noxia ATCC 43541*	ZP_06603766.1	Firmicutes
114_Bsu	296330046	*Bacillus subtilis subsp. spizizenii ATCC 6633*	ZP_06872529.1	Firmicutes
115_Mau	332982541	*Mahella australiensis 50-1 BON*	YP_004463982.1	Firmicutes
116_Lac	325956380	*Lactobacillus acidophilus 30SC*	YP_004291792.1	Firmicutes
117_Smu	397650148	*Streptococcus mutans GS-5*	YP_006490675.1	Firmicutes
118_Aar	302391855	*Acetohalobium arabaticum DSM 5501*	YP_003827675.1	Firmicutes
119_Rce	506407022	*Ruminiclostridium cellulolyticum*	WP_015926741.1	Firmicutes
120_Tbr	332298391	*Treponema brennaborense DSM 12168*	YP_004440313.1	Spirochaetales
121_Saf	383790014	*Spirochaeta africana DSM 8902*	YP_005474588.1	Spirochaetales
122_Sco	330836901	*Sphaerochaeta coccoides DSM 17374*	YP_004411542.1	Spirochaetales
123_Bhy	225619033	*Brachyspira hyodysenteriae WA1*	YP_002720259.1	Spirochaetales
124_Nsp	17232137	*Nostoc sp. PCC 7120*	NP_488685.1	Cyanobacteria
125_Ama	209527099	*Arthrospira maxima CS-328*	ZP_03275613.1	Cyanobacteria
126_Ssp	16332282	*Synechocystis sp. PCC 6803*	NP_443010.1	Cyanobacteria
127_Pma	123968364	*Prochlorococcus marinus str. AS9601*	YP_001009222.1	Cyanobacteria
128_Gvi	37523829	*Gloeobacter violaceus PCC 7421*	NP_927206.1	Cyanobacteria

129_CPr	46445743	*Candidatus Protochlamydia amoebophila UWE25*	YP_007108.1	Chlamydiales
130_Pac	338174863	*Parachlamydia acanthamoebae UV-7*	YP_004651673.1	Chlamydiales
131_Cpe	330444153	*Chlamydophila pecorum E58*	YP_004377139.1	Chlamydiales
132_Cpn	16752433	*Chlamydophila pneumoniae AR39*	NP_444692.1	Chlamydiales
133_Cfe	89898680	*Chlamydophila felis Fe/C-56*	YP_515790.1	Chlamydiales
134_Ctr	301335976	*Chlamydia trachomatis L2tet1*	ZP_07224220.1	Chlamydiales
135_StuS	232164	*Solanum tuberosum (Small Sub.)*		Viridiplantae
136_AthS	1575754	*Arabidopsis thaliana (Small subunit 1)*		Viridiplantae
137_OtaS	308806175	*Ostreococcus tauri*	XP_003080399.1	Viridiplantae
138_OtaL	308814250	*Ostreococcus tauri*	XP_003084430.1	Viridiplantae
139_AthL1	1032280077	*Arabidopsis thaliana (APL1)*		Viridiplantae
140_AthL2	1032299354	*Arabidopsis thaliana (APL2)*		Viridiplantae
141_ZmaSh2	1707924	*Zea mays (Sh2)*		Viridiplantae
142_StuL	232166	*Solanum tuberosum (Large Sub.)*		Viridiplantae
143_AthL3	1032282331	*Arabidopsis thaliana (APL3)*		Viridiplantae
144_AthL4	1032295466	*Arabidopsis thaliana (APL4)*		Viridiplantae

Fig. S6. Sequences of ADP-glucose pyrophosphorylases from plants used to analyze the conservancy of residues. Sequences, GI numbers, and accession codes were obtained as indicated in Materials and Methods.

#	GI number	Accession	Species
1	297812109	XP_002873938.1	*Arabidopsis lyrata subsp. lyrata*
2	297821353	XP_002878559.1	*Arabidopsis lyrata subsp. lyrata*
3	1032280077	OAO94405.1	*Arabidopsis thaliana*
4	1032282331	OAO96658.1	*Arabidopsis thaliana*
5	1032295466	OAP09792.1	*Arabidopsis thaliana*
6	1032299354	OAP13679.1	*Arabidopsis thaliana*
7	1575754	AAB09585.1	*Arabidopsis thaliana*
8	15238933	NP_199641.1	*Arabidopsis thaliana*
9	357116651	XP_003560093.1	*Brachypodium distachyon*
10	357132398	XP_003567817.1	*Brachypodium distachyon*
11	357145851	XP_003573789.1	*Brachypodium distachyon*
12	159467349	XP_001691854.1	*Chlamydomonas reinhardtii*
13	356508352	XP_003522921.1	*Glycine max*
14	356509672	XP_003523570.1	*Glycine max*
15	356517038	XP_003527197.1	*Glycine max*
16	356518710	XP_003528021.1	*Glycine max*
17	356538761	XP_003537869.1	*Glycine max*
18	356545193	XP_003541029.1	*Glycine max*
19	356553863	XP_003545270.1	*Glycine max*
20	356562361	XP_003549440.1	*Glycine max*
21	356563435	XP_003549968.1	*Glycine max*
22	357462397	XP_003601480.1	*Medicago truncatula*
23	357473317	XP_003606943.1	*Medicago truncatula*
24	357495273	XP_003617925.1	*Medicago truncatula*
25	255070935	XP_002507549.1	*Micromonas commoda*
26	255080070	XP_002503615.1	*Micromonas commoda*
27	303271247	XP_003054985.1	*Micromonas pusilla CCMP1545*
28	303273364	XP_003056043.1	*Micromonas pusilla CCMP1545*
29	145356323	XP_001422382.1	*Ostreococcus lucimarinus CCE9901*
30	1199289822	OUS44927.1	*Ostreococcus tauri*
31	1199292237	OUS47325.1	*Ostreococcus tauri*
32	145349062	XP_001418959.1	*Ostreococus lucimarinus CCE9901*
33	224062107	XP_002300758.1	*Populus trichocarpa*
34	224100249	XP_002311802.1	*Populus trichocarpa*
35	224103389	XP_002313036.1	*Populus trichocarpa*
36	255538708	XP_002510419.1	*Ricinus communis*
37	255543725	XP_002512925.1	*Ricinus communis*
38	255552303	XP_002517196.1	*Ricinus communis*
39	302783933	XP_002973739.1	*Selaginella moellendorffii*
40	232164	P23509.2	*Solanum tuberosum*

41	232166	Q00081.1	*Solanum tuberosum*
42	242033053	XP_002463921.1	*Sorghum bicolor*
43	242048788	XP_002462140.1	*Sorghum bicolor*
44	242053733	XP_002456012.1	*Sorghum bicolor*
45	242088961	XP_002440313.1	*Sorghum bicolor*
46	225432564	XP_002281069.1	*Vitis vinifera*
47	225447450	XP_002263255.1	*Vitis vinifera*
48	225458219	XP_002281223.1	*Vitis vinifera*
49	225428422	XP_002283855.1	*Vitis vinifera*
50	302840808	XP_002951950.1	*Volvox carteri f. nagariensis*
51	302849075	XP_002956068.1	*Volvox carteri f. nagariensis*
52	1707924	P55241.1	*Zea mays*
53	162460455	NP_001106017.1	*Zea mays*
54	162461970	NP_001105038.1	*Zea mays*
55	162462257	NP_001105178.1	*Zea mays*
56	162463875	NP_001106058.1	*Zea mays*
57	189027076	NP_001121104.1	*Zea mays*

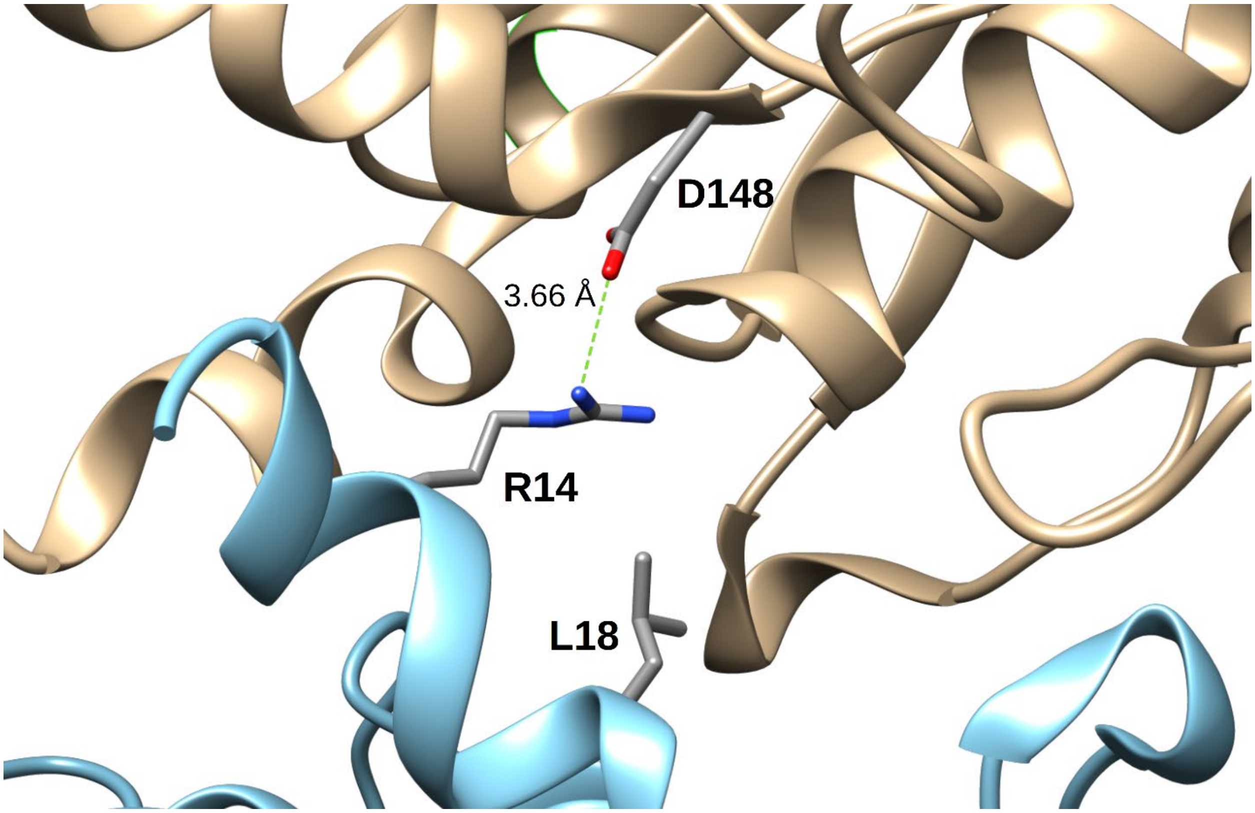

Fig. S7. Dimer interaction between D148 and R14 in the *E. coli* ADP-Glc PPase.
Residues D148 and L18 are in homologous position to D141 and R11 in *A. tumefaciens* ADP-Glc PPase, respectively. Figure has been constructed using the coordinates from the *E. coli* ADP-Glc PPase structure (PDB: 5L6S) as indicated in Materials and Methods.

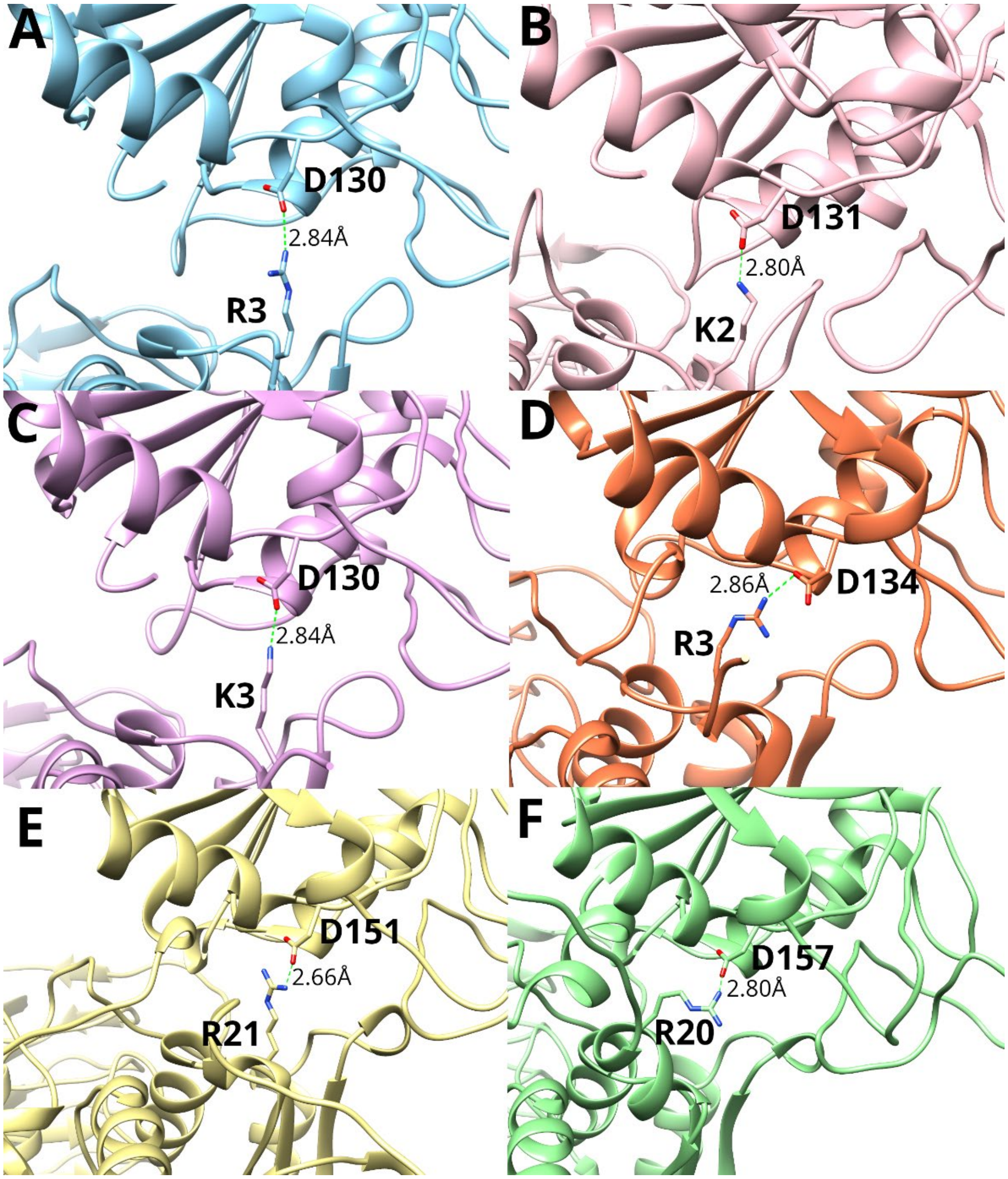

	1_Atu	*Agrobacterium tumefaciens*	----------------MSEKRVQPLA**R**DAMAYVLAGG	ILAGDHIYKM**D**YEYMLQQHVDS
	63_Eco	*Escherichia coli*	---------MVSLEKNDHLMLA**R**QLPLKSVALILAGG	ILAGDHIYKQ**D**YSRMLIDHVEK
	135_StuS	*Solanum tuberosum (small)*	--------MAVSDSQNSQTCLDPDAS**R**SVLGIILGGG	ILAGDHLYRM**D**YEKFIQAHRET
**A**	110_Vin	*Vulgatibacter incomptus*	-------------------------MS**R**TLALILAGG	VFGADHVYRM**D**VRQMLHFHRET
**B**	124_Nsp	*Nostoc sp.*	-------------------------M**K**KVLAIILGGG	ILSGDHLYRM**D**YRLFIQRHRET
**C**	111_Cil	*Corallococcus llansteffanensis*	-------------------------MS**K**LLAMILAGG	VFGADHVYRM**D**VRKMLDFHIER
**D**	106_Sco	*Streptomyces coelicolor*	---------------------MR**R**GGPSVLGIVLAGG	VFGADHVYRM**D**PRQMLAQHIES
**E**	53_Neu	*Nitrosomonas europaea*	------MKVQPAVQTNDNPRFVSTLT**R**NTLALILAGG	ILGGDHIYKM**D**YGRILAEHVER
**F**	40_Nsp	*Nitrosomonas sp*	MDKTKLTHAQSQAEYKSSS**R**FHSNISHETLALILAGG	ILGGDHIYKM**D**YSKLLAEHIEK

Fig. S8. Modeling of the N-terminal domain interaction in representative ADP-glucose pyrophosphorylases

Enzymes in panel A to F were selected to represent different type of possible interactions in different species. These interactions are with the Asp homologous to Asp141 in *A. tumefaciens* Enzyme. The sequences are also aligned to the structures from *A. tumefaciens*, *E. coli*, and *S. tuberosum* of which crystal structures are displayed in Fig. 1 and Fig. S7. Arrows in the bottom table represent the homologous positions to the Arg11 and Asp141 in *A. tumefaciens*.

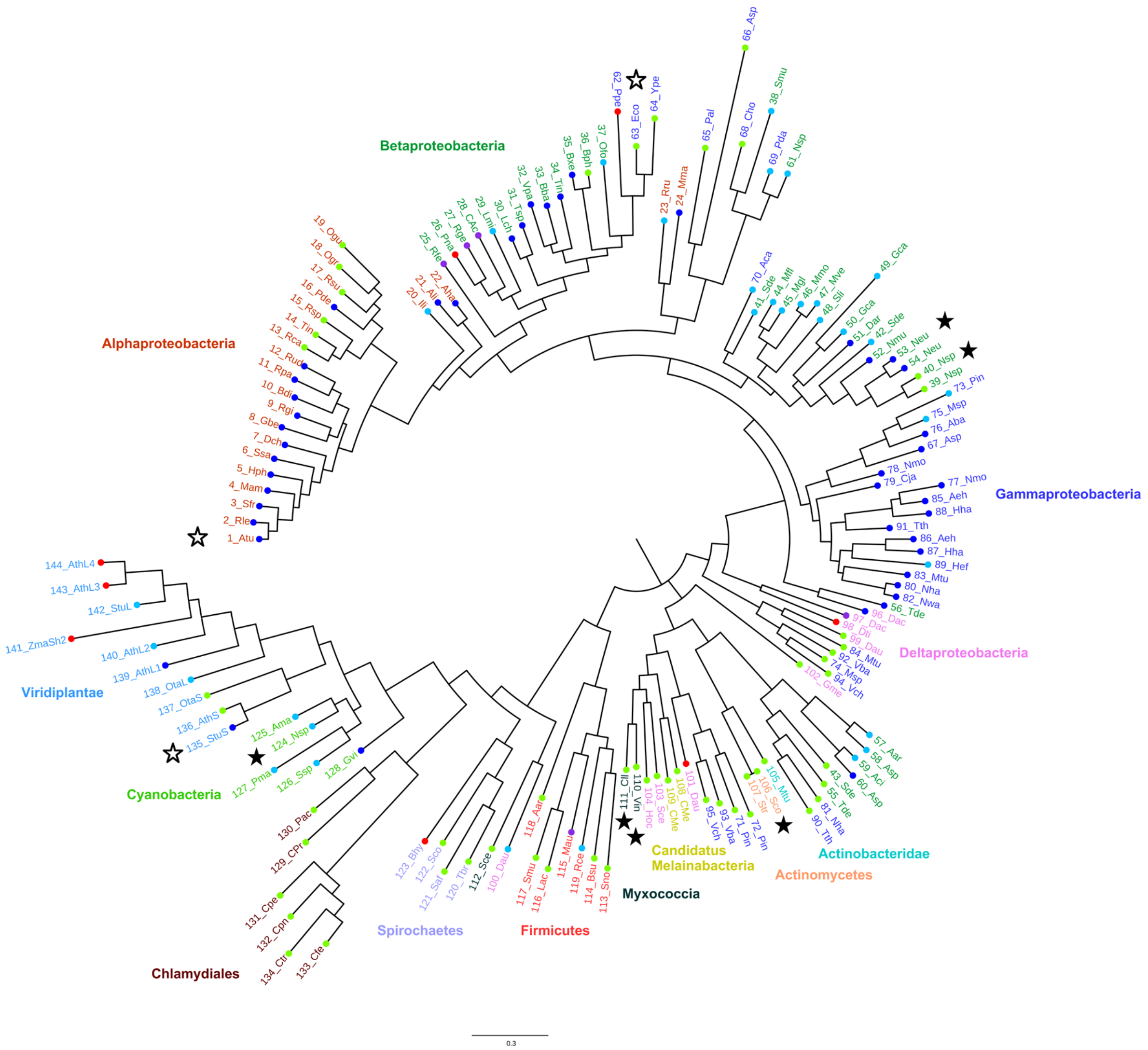

Fig. S9. Phylogenetic tree of ADP-glucose pyrophosphorylases from bacteria and photosynthetic eukaryotes. Sequences of the glgC gene products from bacteria and homologous from photosynthetic eukaryotes were collected and the tree was built as described in Materials and Methods. In the tree, it is the taxonomy information matching colors with the id codes for each individual gene. Blue circles indicate enzymes with Arg and Asp in homologous positions 11 and 141 respectively (*A. tumefaciens nomenclature*). Light blue circles have Lys and Asp, Green does not have an Arg or Lys in position 11, but has Asp in position 141. Purple has an Arg in position 11, but no Asp in 141. Red has neither of those residues conserved. White stars depict variants with crystal structures analyzed in this work. Black stars are forms that were modeled in Fig. S8.

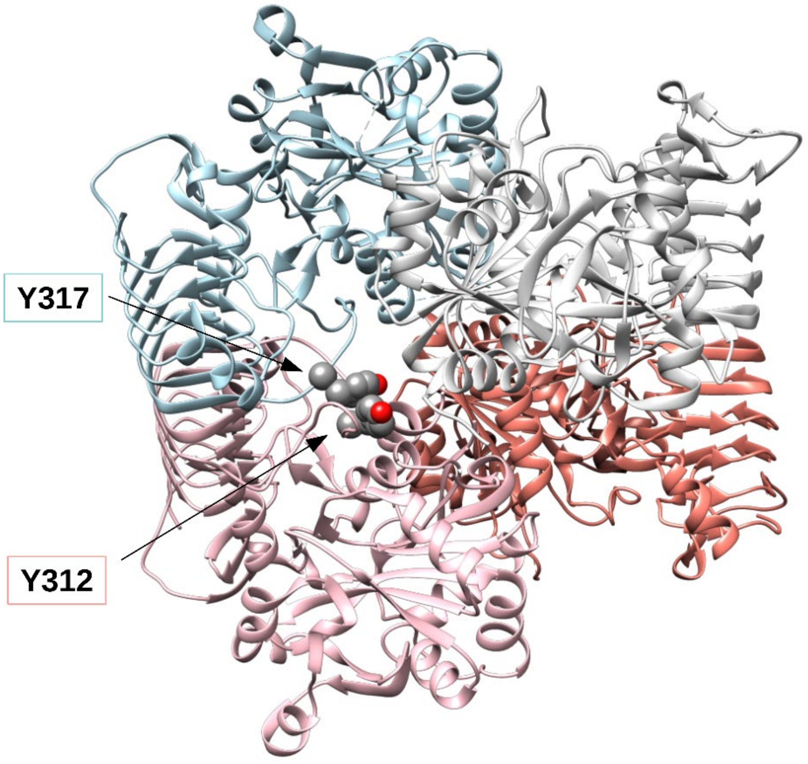

Fig. S10. Interaction at the CC interface of the homotetrameric potato tuber small subunit ADP-Glc PPase. Depicted residues Tyr317 from the light blue subunit interact with the Tyr312 subunit in pink. The interaction is “edge to face” as described in Discussion.
