# Supplementary material for: A critical inter‐subunit interaction for the transmission of the allosteric signal in the Agrobacterium tumefaciens ADP‐glucose pyrophosphorylase
Source: Protein Sci. 2023 Sep 1;32(9):e4747. doi: 10.1002/pro.4747 (PMC10461462; doi:10.1002/pro.4747)
Supplement: Supplementary file 2 — Table S1. Kinetic parameters for the activators of the A. tumefaciens ADP‐Glc PPase wild type, and mutants. Table S2. Kinetic parameters for the substrate (ATP) of ADP‐Glc PPase wild type, and mutants. Table S3. Conservancy of residues Arg11 and Asp141 in various plant and bacterial ADP‐glucose pyrophosphorylases. [file PRO-32-e4747-s002.pdf]

Supplementary material
(Tables)

Table S1. Kinetic parameters for the activators of the *A. tumefaciens* ADP-Glc PPase wild type, and mutants.

Enzyme [a]	Fru6P				Pyruvate			
	$A_{0.5}$ (mM)	n_H	V_m (U/mg)	Activation [b] (V_m/V_0) (-fold)	$A_{0.5}$ (mM)	n_H	V_m (U/mg)	Activation [b] (V_m/V_0) (-fold)
WT	0.34 ± 0.07	2.1 ± 0.5	105.2 ± 3.9	9.01	0.45 ± 0.04	2.1 ± 0.4	87.4 ± 2.0	8.53
D141E	0.03 ± 0.01	1.9 ± 0.6	31.86 ± 0.17	1.81	0.06 ± 0.03	1.2 ± 0.5	21.73 ± 0.23	1.47
D141A	0.84 ± 0.07	2.7 ± 0.6	1.13 ± 0.04	3.32	0.30 ± 0.02	2.8 ± 0.5	7.29 ± 0.14	17.8
D141N	2.97 ± 0.15	3.9 ± 0.6	0.02 ± 0.01	1.20	N/A[d]	N/A[d]	0.02 ± 0.01	1.00
D141R	N/A[d]	N/A[d]	0.02 ± 0.01	0.96	N/A[d]	N/A[d]	1.01 ± 0.17	0.95
R11K	0.05 ± 0.02	1.0 ± 0.3	47.73 ± 0.94	2.91	0.04 ± 0.02	0.6 ± 0.3	36.9 ± 2.3	1.86
R11A[c]	0.030 ± 0.008	1.3 ± 0.4	7.2 ± 0.2	1.8	N/A[d]	N/A[d]	5.0 ± 0.5	1
R11D	N/A[d]	N/A[d]	9.96 ± 0.16	0.98	N/A[d]	N/A[d]	9.96 ± 0.55	0.98
R11D/D141R	1.32 ± 0.88	1.5 ± 0.3	13.75 ± 0.96	1.25	N/A	N/A	10.49 ± 0.87	0.99

[a] Assays were performed as described in Materials and Methods under "activator saturation assay". ATP concentration was 1.5 mM.
[b] Activation fold is calculated by dividing the maximum velocity (V_m) by the velocity in the absence of activator (V_0).
[c] The results for the R11A mutant of the ADP-Glc PPase from *A. tumefaciens* were taken from literature as described in Materials and Methods.
[d] No significant activation was observed to calculate activation parameters.

Table S2. Kinetic parameters for the substrate (ATP) of ADP-Glc PPase wild type, and mutants

Enzyme [a]	Control			+ Fru6P [c]			+ Pyr [c]		
	$S_{0.5}$ (ATP) (mM)	n_H	V_m (U/mg)	$S_{0.5}$ (ATP) (mM)	n_H	V_m (U/mg)	$S_{0.5}$ (ATP) (mM)	n_H	V_m (U/mg)
WT	0.21 ± 0.01	1.9 ± 0.2	11.18 ± 0.30	0.08 ± 0.01	1.7 ± 0.1	123.5 ± 2.4	0.13 ± 0.01	2.2 ± 0.2	85.4 ± 1.9
D141E	0.24 ± 0.02	1.8 ± 0.2	12.58 ± 0.23	0.09 ± 0.01	1.4 ± 0.1	27.15 ± 0.40	0.10 ± 0.03	2.2 ± 1.0	21.76 ± 0.59
D141A	1.49 ± 0.06	2.0 ± 0.2	3.82 ± 0.15	1.39 ± 0.09	2.1 ± 0.3	3.52 ± 0.11	0.66 ± 0.03	2.9 ± 0.3	8.28 ± 0.11
D141N	3.99 ± 0.70	3.1 ± 1.1	0.060 ± 0.010	3.01 ± 0.39	3.0 ± 0.9	0.050 ± 0.010	3.19 ± 0.21	4.1 ± 0.9	0.050 ± 0.010
D141R	0.56 ± 0.10	1.0 ± 0.2	0.020 ± 0.010	0.70 ± 0.24	0.8 ± 0.2	0.030 ± 0.003	0.32 ± 0.02	1.1 ± 0.1	0.020 ± 0.010
R11K	1.01 ± 0.05	1.4 ± 0.1	40.86 ± 0.98	0.06 ± 0.03	1.2 ± 0.5	47.1 ± 1.9	0.23 ± 0.01	2.8 ± 0.5	36.21 ± 0.64
R11A[b]	0.47 ± 0.10	2.4 ± 0.5	4.10 ± 0.50	0.05 ± 0.01	1.5 ± 0.5	8.30 ± 0.50	0.29 ± 0.02	2.1 ± 0.4	5.00 ± 0.30
R11D	1.11 ± 0.25	1.0 ± 0.1	15.0 ± 1.3	3.39 ± 1.81	0.7 ± 0.1	20.7 ± 4.0	1.13 ± 0.34	0.8 ± 0.1	16.72 ± 1.61
R11D/D141R	0.44 ± 0.03	1.7 ± 0.2	12.11 ± 0.31	0.06 ± 0.01	1.4 ± 0.7	10.29 ± 0.13	0.18 ± 0.01	2.4 ± 0.4	13.85 ± 0.19

[a] Assays were performed as described in substrate saturation assay under Materials and Methods.
[b] The results for R11A mutant ADP-Glc PPase for *A. tumefaciens* were taken from the literature as described in Materials and Methods
[c] Concentration of the activator was 1.5 mM

Table S3. Conservancy of residues Arg11 and Asp141 in various plant and bacterial ADP-glucose pyrophosphorylases.

Sequence similarities for conserved residue Arg11, and Asp141			
**Plants (Asp141)**		**Bacteria (Asp141)**	
Amino acid	Percentage*	Amino acid	Percentage*
Asp (D)	86.0%	Asp (D)	93.3%
Asn (N)	14.0%	Asn (N)	6.0%
		Glu (E)	0.7%
**Plants (Arg11)**		**Bacteria (Arg11)**	
Amino acid	Percentage*	Amino acid	Percentage*
Arg (R)	21.0%	Arg (R)	36.6%
Lys (K)	38.6%	Lys (K)	23.9%
D, E, N & other	40.4%	D, E, N, Y, F, A, H, Q, G	39.5%

* The percentage was calculated by using the final number sequences as a 100%. For plants, we used 57 sequences, whereas for bacteria we used 134 sequences without any duplicates.
